# A reference single-cell regulomic and transcriptomic map of cynomolgus monkeys

**DOI:** 10.1038/s41467-022-31770-x

**Published:** 2022-07-13

**Authors:** Jiao Qu, Fa Yang, Tao Zhu, Yingshuo Wang, Wen Fang, Yan Ding, Xue Zhao, Xianjia Qi, Qiangmin Xie, Ming Chen, Qiang Xu, Yicheng Xie, Yang Sun, Dijun Chen

**Affiliations:** 1 State Key Laboratory of Pharmaceutical Biotechnology, School of Life Sciences, Nanjing University, 210023 Nanjing, China; 2 The Children’s Hospital, Zhejiang University School of Medicine, National Clinical Research Center for Child Health, 310052 Hangzhou, China; 3 Shanghai XuRan Biotechnology Co., Ltd., 1088 Zhongchun Road, 201109 Shanghai, China; 4 College of Life Sciences, Zhejiang University, 310058 Hangzhou, China; 5 Chemistry and Biomedicine Innovation Center (ChemBIC), Nanjing University, 210023 Nanjing, China

**Keywords:** Biological models, Functional genomics, Data acquisition, RNA sequencing

## Abstract

Non-human primates are attractive laboratory animal models that accurately reflect both developmental and pathological features of humans. Here we present a compendium of cell types across multiple organs in cynomolgus monkeys (*Macaca fascicularis*) using both single-cell chromatin accessibility and RNA sequencing data. The integrated cell map enables in-depth dissection and comparison of molecular dynamics, cell-type compositions and cellular heterogeneity across multiple tissues and organs. Using single-cell transcriptomic data, we infer pseudotime cell trajectories and cell-cell communications to uncover key molecular signatures underlying their cellular processes. Furthermore, we identify various cell-specific *cis*-regulatory elements and construct organ-specific gene regulatory networks at the single-cell level. Finally, we perform comparative analyses of single-cell landscapes among mouse, monkey and human. We show that cynomolgus monkey has strikingly higher degree of similarities in terms of immune-associated gene expression patterns and cellular communications to human than mouse. Taken together, our study provides a valuable resource for non-human primate cell biology.

## Abstract (web summary)

Non-human primates are attractive laboratory animal models that can accurately reflect some developmental and pathological features of humans. Here the authors chart a reference cell map of cynomolgus monkeys using both scATAC-seq and scRNA-seq data across multiple organs, providing insights into the molecular dynamics and cellular heterogeneity of this organism.

## Introduction

Non-human primates (NHP) are phylogenetically close to humans and show various human-like characteristics, including genetics, organ development, physiological function, pathological response and biochemical metabolism. Hence NHP are extremely valuable as experimental animal models for medical research and drug development^1^. *Macaca fascicularis* (cynomolgus monkeys) are such excellent NHP models for biomedical research^2^. Since cells are the fundamental unit of all life, direct comparison of cell identities and cell-type compositions in organs with a similar function between organisms would help to transfer knowledge in primates to medical research. In this regard, it is of vital importance to understand the cellular composition and heterogeneity of organs in the primate model like cynomolgus monkeys.

Rapid advances in single-cell multi-omics technologies have enabled molecular quantification of thousands of cells at once, leading to meticulous insight into organ compositions and molecular mechanisms driving cellular heterogeneity^3^. Previous studies^4–8^ have mapped the single-cell landscapes across multiple organs in humans and mice, expanding our knowledge about the cellular heterogeneity underlying normal development and aging. Three-dimensional multicellular culture systems combined with single-cell transcriptome sequencing technology enable to chart the cellular and molecular dynamic changes during organ growth and development^9–11^. In addition, extensive efforts^10–13^ have been achieved to investigate how cells are perturbed in various disease conditions, including cancer and neurological disorders.

Mice have long been used as a representative model organism for mammalian development and physiology. Recently, extensive comparative analyses based on single-cell transcriptomics data have shown that both cell types and associated gene regulatory networks are conserved between human and mouse^4,14–23^, which provides a new perspective for disease mechanism interpretation and intervention. However, it has been widely recognized that there are significant differences between mice and humans in terms of development and physiology^24^. For example, single-cell transcriptomic analysis revealed remarkable similarities and differences in lineage marker-associated gene expression and regulatory networks during spermatogenesis^19^ and embryogenesis^21^ between human, cynomolgus, and mouse. In a recent study, comparison of cell subsets of colon neurons in the human and mouse enteric nervous system (ENS) revealed differences on the basis of transcriptional programs and neuro-immune interactions^22^. The mapped differences may reflect divergent adaptations to feeding behavior by ENS between human and mouse. Besides, although the architecture and regulatory role of the immune system is conserved between mouse and human, it is notoriously difficult to translate the immunological principles from laboratory mice to humans^2^, partially due to their basic immunological differences^25^ and/or the immunological immaturity of the laboratory mouse^26^. In short, these observed differences may limit the immediate translational value of findings from the mouse model to biomedical research.

Primate experiments are more valuable as they can better simulate human diseases and promote scientific research owing to high genetic similarity between primates and humans^27^. Although the potential importance and values of NHP models in basic research are indispensable, an organism-wide single-cell atlas is still pending for primates.

Here, we present a compendium of single-cell regulomic and transcriptomic data in cynomolgus monkeys that comprises 40 distinct cell types from 16 representative organs and tissues, greatly extending our current view^28–30^ of single-cell landscapes in this model species. This cell atlas—which we denote ‘Monkey Atlas’—represents a new resource for cynomolgus monkey cell biology.

## Results

### Mapping a cynomolgus monkey multi-organ cell atlas by multi-omic analysis

**Fig. 1:**
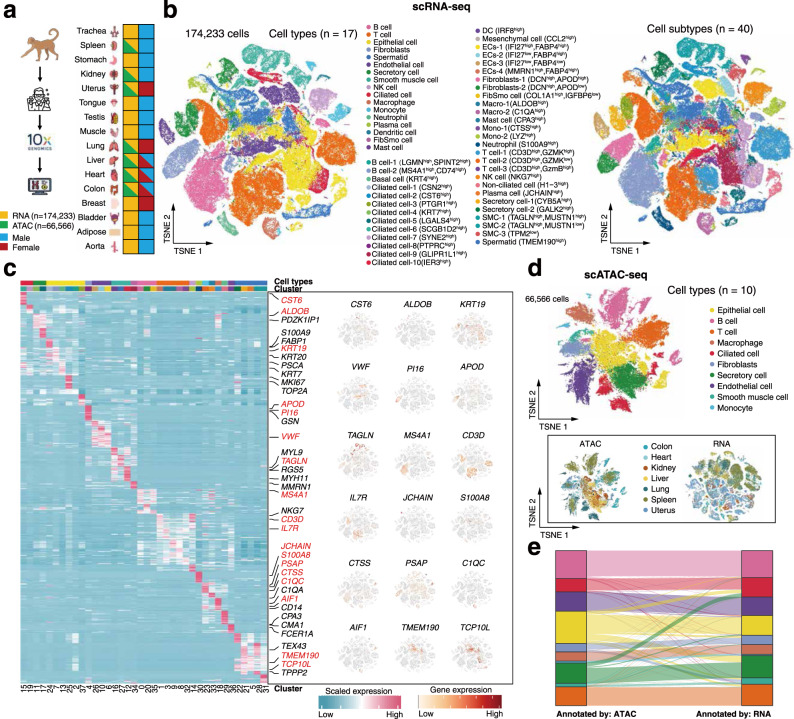
Single-cell landscapes across 16 organs in cynomolgus monkeys. **a** Schematic illustration of the workflow. Single-cell transcriptome (scRNA-seq) analysis was conducted in 16 organs and chromatin accessibility (scATAC-seq) analysis in seven organs from adult cynomolgus monkeys. **b** Cell type identification based on scRNA-seq data. In total 17 major cell types and 40 cell subtypes were identified. T-SNE visualizations of cells were colored either by the major cell types (left) or by the cell subtypes (right). **c** Heatmap showing the scaled expression levels of cell type-specific marker genes (left). Expression patterns of 18 representative marker genes are shown on the right. **d** T-SNE illustration of cell type annotation based on scATAC-seq data analysis. Ten major cell types were identified from 66,566 cells. Cells were colored by major cell types (the top panel) or by organs (bottom left) in the t-SNE plots. The bottom right t-SNE plot shows the clustering of cells based on scRNA-seq data for the seven organs with matched RNA-ATAC samples. **e** Sankey diagram showing the consistence of cell type annotations between scRNA-seq and scATAC-seq data. Only the seven organs with matched RNA-ATAC data were used for the analysis. The color code refers to (**d**).

To generate a reference cell map of monkey, we performed both single-cell RNA sequencing (scRNA-seq; 10x Genomics; *n* = 174,233 cells) and scATAC-seq (10x Genomics; *n* = 66,566 cells) in 16 tissues and organs from one male or/and one female cynomolgus monkeys (Fig. 1a, Supplementary Fig. 1 and Supplementary Table 1). We integrated all of the scRNA-seq data using canonical correlation analysis (CCA)^31^ to correct for potential batch effects. Unsupervised clustering based on t-distribution stochastic neighbor embedding (t-SNE) resolved major cell types, including epithelial, ciliated epithelial, mesenchymal, immune, endothelial, spermatid, and secretory cell populations. These cells could be annotated as 40 transcriptionally distinct clusters with cluster-specific markers (Fig. 1b, c and Supplementary Table 2). Due to technical and financial constraints, not every organ was analyzed in each monkey or by both data modalities. Nevertheless, the overall gene expression patterns and cell compositions for the same organ or functional related organs (e.g., stomach, liver, spleen, and colon from the digestive system) are generally consistent (Supplementary Figs. 2, 3), highlighting the reproducibility of our data. In particular, the analysis of multiple organs from the same monkey enables us to obtain data that are controlled for uncertain effects (such as age, sex, diet, environment, and so on).

The scATAC-seq experiment was performed in relatively few organs (*n* = 7), including liver, colon, uterus, spleen, lung, heart, and kidney. To define cell types based on scATAC-seq data in these organs, we created a count matrix of fragments across the genome. We demonstrated the overall high quality of scATAC-seq data based on the enrichment analysis of accessible DNA sequences relative to the transcriptional start site (TSS) and the size distribution of unique fragments (Supplementary Fig. 4). After batch effect correction with Harmony^32^ as implemented in ArchR^33^ (Supplementary Fig. 5a), t-SNE clustering analysis of the integrated scATAC-seq data revealed ten major cell types (Fig. 1d), which were annotated based on chromatin accessibility at the promoter regions of well-characterized marker genes (Supplementary Fig. 6).

To confirm the consistency of scATAC-seq and scRNA-seq data for cell type annotation, we first performed cross-modality integration analysis in organ-matched samples between RNA and ATAC (*n* = 7 organs) using a mutual nearest neighbors (MNNs) approach (Supplementary Fig. 5b–e; see “Methods”). We then assigned the cell type of each clusters using labels annotated from scRNA-seq data and identified twelve major cell types in the RNA-ATAC integration (Supplementary Fig. 5f). All major cell types were successfully recovered except the plasma cell (Supplementary Fig. 5b, f). In this manner, cell types of ATAC clusters can be predicted from RNA clusters on the basis of cross-modality integration (Supplementary Fig. 5g). We found that cell types identified by scRNA-seq and scATAC-seq data are highly consistent (Fig. 1e), with ten ATAC clusters one-to-one linked to RNA clusters (Supplementary Fig. 5h). Collectively, the above results highlight the quality of the dataset.

### Heterogeneity and dynamics of main cell components across organs

**Fig. 2:**
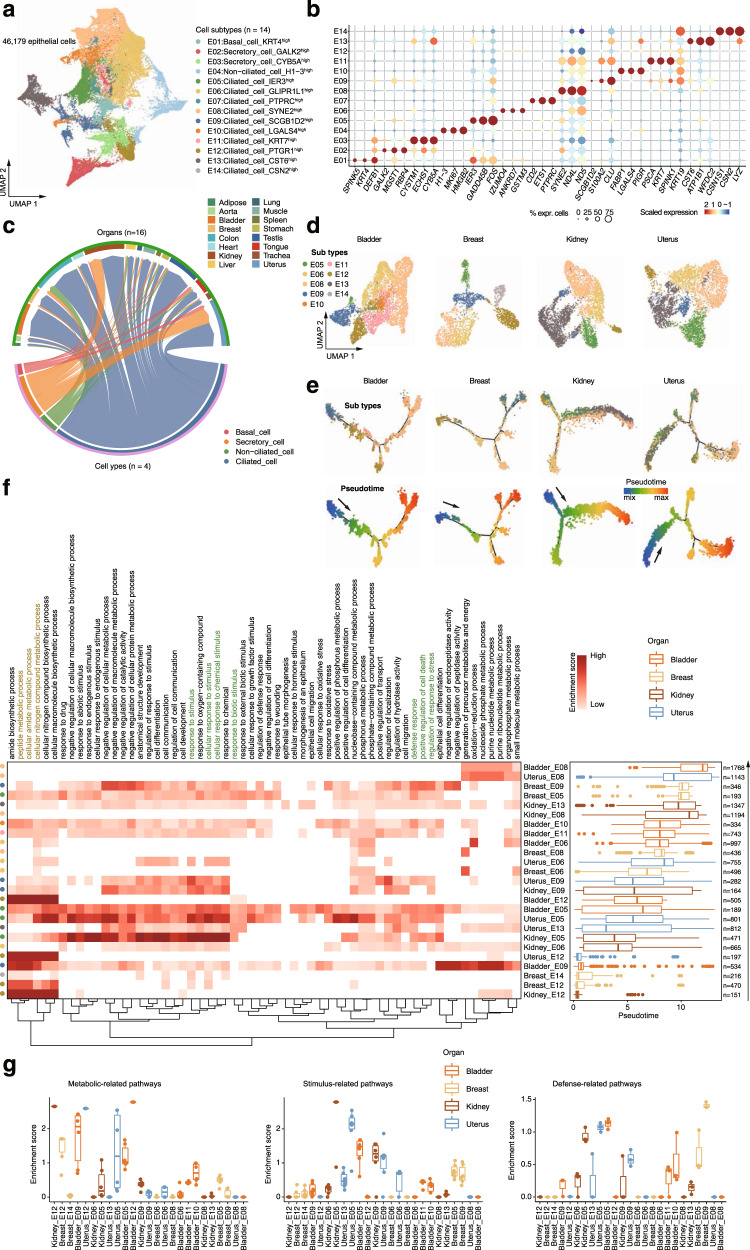
The heterogeneity and developmental state of epithelial cells. **a** Distribution of 14 epithelial cell subtypes on the UMAP. **b** Dot plot shows representative marker genes across the epithelial subtypes. The dot size is proportional to the fraction of cells which express specific genes. The color scale corresponds to the relative expression of specific genes. **c** Chord diagram showing the mapping of cells between major cell types and organs. The width of the arrow represents the proportion of cells in a given cell type. **d** UMAP showing the distribution of ciliated cell subtypes in bladder, breast, kidney and uterus. **e** Monocle2 detects semi-supervised pseudo-temporal trajectories of ciliated cell subtypes in bladder, breast, bladder and uterus. The trajectory is colored by cell subtypes (top) or pseudotime (bottom). **f** Heatmap showing the enrichment of pathways in epithelial subtypes of different organs based on GSEA analyses. Box plots show the distribution of estimated pesudotime of epithelial cells by Monocle2. The boxes indicate the 25% quantile, median (horizontal line), 75% quantile and Tukey-style whiskers (beyond the box). **g** Box plots showing the enrichment scores of metabolic-related pathways (*n* = 3), stimulus-related pathways (*n* = 4), and defense-related pathways (*n* = 3) in different epithelial subtypes of different organs. Each point indicates a specific pathway. The boxes indicate the 25% quantile, median (horizontal line), 75% quantile.

Epithelial cells account for the largest part in the integrated transcriptomic cell map. To dissect epithelial heterogeneity, we extracted epithelial cells and performed unsupervised sub-clustering analysis (Fig. 2a). The analysis identified 14 epithelial cell clusters (E01-E14), including basal cells, secretory cells, ciliated cells and non-ciliated cells, according to the unique pattern of marker gene expression (Fig. 2a, b). As expected, secretory cells were mostly found in epidermis or parenchymatous organs such as spleen, kidney, colon and uterus (Fig. 2c). It is worth noting that there are a large proportion of ciliated epithelial cells in various tissues (Fig. 2c and Supplementary Fig. 7a, b). To explore the developmental and functional dynamics of ciliated epithelial cell subtypes, we performed pseudotime trajectory analysis using both Monocle^34^ and RNA velocity^35^ in organs with relatively more ciliated epithelial cells, including bladder, breast, kidney and uterus (Fig. 2d). We observed similar developmental trajectories of ciliated epithelial cells in the investigated organs, differentiation from a progenitor-like cell state to a mature state (Fig. 2e and Supplementary Fig. 7c), suggesting that ciliated epithelial cells may share a common differentiation trajectory in different organs. Accordingly, highly expressed genes along the pseudotime were enriched in gene ontology (GO) terms related to metabolic processes, cellular response to stimulus and defense response in a sequential manner (Fig. 2f, g). The analysis is in agreement with the knowledge that ciliated epithelial cells play an important role in cleaning pathogenic microorganisms and signal transduction^36^.

**Fig. 3:**
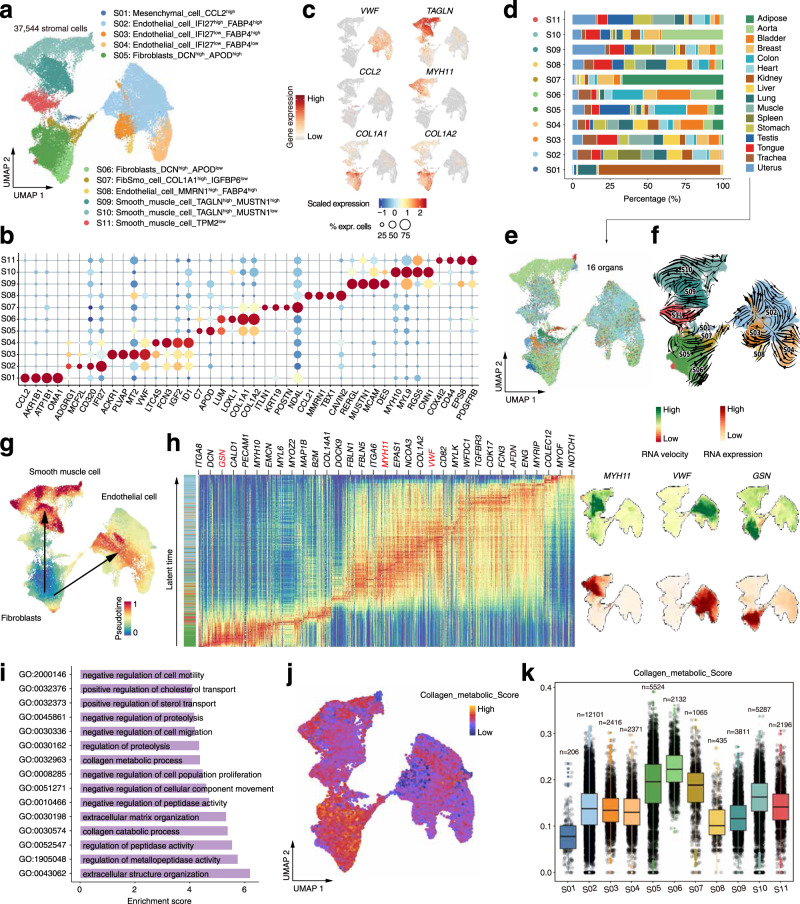
Heterogeneity of stromal cells in different organs. **a** Distribution of eleven stromal cell subtypes on the UMAP. **b** Dot plots showing representative marker genes across the stromal subtypes. Dot size is proportional to the fraction of cells expressing specific genes. Color intensity corresponds to the relative expression of specific genes. **c** Feature plot showing the expression of selected marker genes. **d** Bar plot showing the percentage of cell subtypes in each organ. **e** Distribution of stromal cells from different organs on the UMAP. **f** Unsupervised pseudotime trajectory of the subtypes (S01-S11) of stromal cells by RNA velocity analysis. Trajectory is colored by cell subtypes. The arrow indicates the direction of cell pseudo-temporal differentiation. **g** UMAPs showing the pseudotime differentiation trajectories of fibroblasts, smooth muscle cells and endothelial cells respectively. **h** Heatmap showing the scaled expression levels of cell-type-specific marker genes along the pseudotime differentiation trajectory. Examples of marker expression are shown in the right UMAPs. **i** Barplot showing the enrichment of functional pathways in fibroblasts (cluster S06). **j**, **k** UMAP plot and Box plot showing the distribution of gene signature scores estimated by UCell based on annotated genes (*n* = 24) from the collagen metabolic pathway. The boxes indicate the 25% quantile, median (horizontal line), 75% quantile and Tukey-style whiskers (beyond the box).

Stromal cells are an important component of body tissues^37^. In the stromal compartment, we identified 11 clusters (S01-S11) belonging to four major cell types, including endothelial cells, fibroblasts, FibSmo cells and smooth muscle cells (Fig. 3a–c and Supplementary Fig. 8a). Although these cell clusters were generally identified in all organs, the heterogeneity of stromal cells was observed in different organs (Fig. 3d, e and Supplementary Fig. 8b). Most mesenchymal cells were generated from kidney; almost all fibroblasts in testis were from S05 (DCN^high^APOD^high^ fibroblasts); there were a large number of TAGLN^high^MUSTN1^low^ smooth muscle cells in the aorta organ but few other smooth muscle cells were enriched in this organ (Fig. 3d). Considering that stromal cells have a certain differentiation potential^38^, we applied RNA velocity analysis to explore developing states of stromal cells (Fig. 3f). The results suggest that fibroblasts might have the capacity of differentiation to smooth muscle cells and endothelial cells (Fig. 3g, h and Supplementary Fig. 8c–e). GO enrichment analysis revealed that fibroblasts highly expressed genes involved in collagen metabolic or collagen catabolic processes (Fig. 3i). In fact, the cluster S06 may be response for the collagen metabolic function since cells in this cluster highly expressed collagen-associated genes (Supplementary Fig. 8f) including type I collagen genes *COL1A1* and *COL1A2* (Fig. 3b). Accordingly, gene signature scores of the collagen metabolic pathway were significantly higher (Mann–Whitney test, *p*-value < 0.05) in S06 than other cell clusters (Supplementary Fig. 8g). Furthermore, to explore the difference of the metabolic capacity of the fibroblasts cluster S06 in different organs, we analyzed the expression pattern and signature scores of genes from the collagen metabolic pathway in the 16 different organs (Supplementary Fig. 8f, h). We found that organs such as adipose, aorta, uterus, bladder, and colon showed relatively higher expression of collagen metabolic genes as well as gene signature scores than other organs, suggesting that the metabolic capacity of fibroblasts (S06) varies in different tissues. Therefore, we speculate that the high potential metabolic capacity of fibroblasts (S06) may help to synthesize and to secrete collagen.

**Fig. 4:**
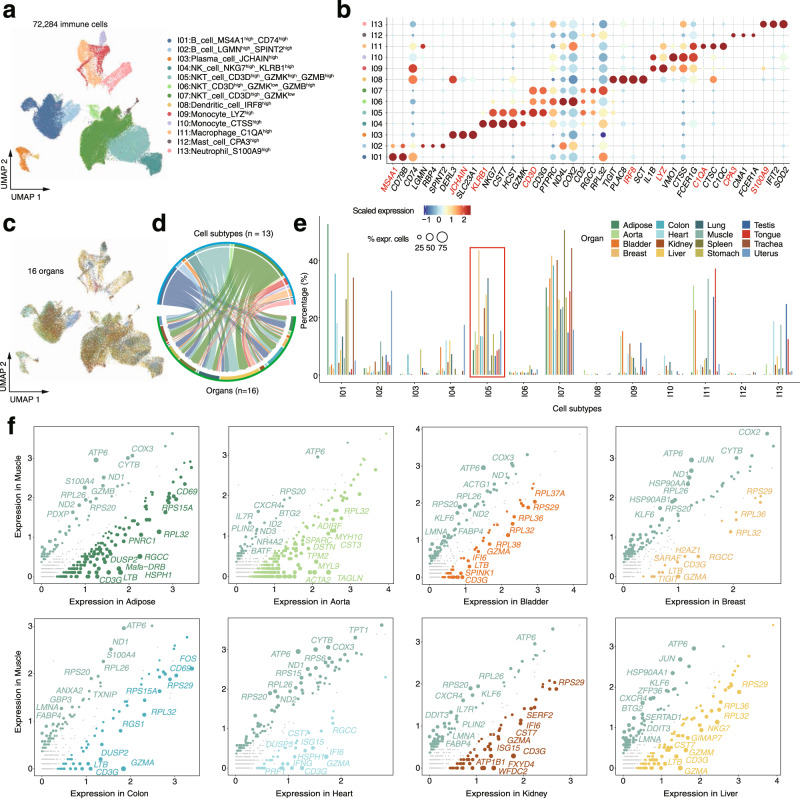
Immune cell heterogeneity in cynomolgus monkeys. **a** Distribution of 13 major immune cell types on the UMAP. **b** Dot plots shows representative top marker genes across the immune subtypes. **c** Distribution of immune cells from different organs on the UMAP. **d** Chord diagram mapping cells between cell types and organs. The width of the arrow represents the proportion of cell types. **e** Bar chart showing the proportion of cells from different organs for each cell subtype. **f** Scatter plots showing differentially expressed genes (DEGs) between muscle and other organs. Each dot represents a DEG, and its size is proportional to the fold change.

Immune cells are essential for maintaining body homeostasis^39^. We identified 72,284 immune-related cells from the investigated organs, including B cells, T cells, and myeloid cells. These cells were further grouped into 13 major clusters (I01-I13) based on known or novel gene signatures (Fig. 4a, b and Supplementary Fig. 9a, b). Although the annotated immune cell clusters can be found in all organs (Fig. 4c, d), the relative proportion of immune cells varied greatly in different organs. For example, we noticed that the proportion of NKT_cell_CD3D^high^_GZMK^high^_GZMB^high^ cells largely varied between muscle and other organs (Fig. 4e). We therefore analyzed the differentially expressed genes between muscle and other organs in the NKT_cell_CD3D^high^_GZMK^high^_GZMB^high^ cells. We observed that mitochondria-related genes (*ATP6*, *COX3* and *ND1*) were top differentially expressed genes in common among all the pairwise comparisons (Fig. 4f and Supplementary Fig. 9c). This suggests that the NKT_cell_CD3D^high^_GZMK^high^_GZMB^high^ cells may have an energy metabolism function^40^.

### Dynamics of cell–cell interactome

**Fig. 5:**
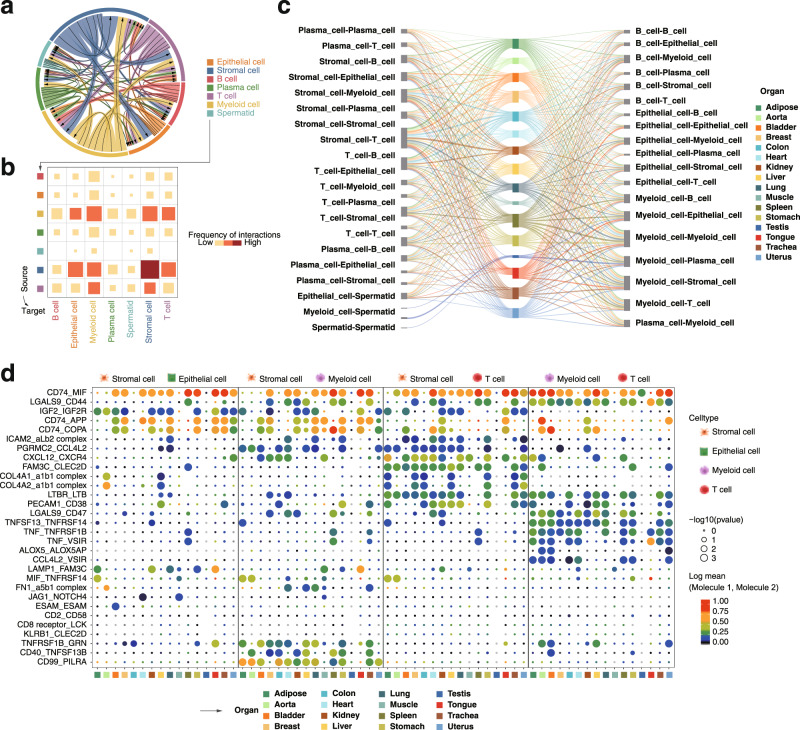
Dynamics of cell–cell communication networks. **a** Chordal diagram of the integrated cell–cell interaction network among the major cell types. **b** Heatmap showing the interaction intensity of cellular interactome from (**a**). Block sizes and colors are proportional to the interaction frequency. **c** Sankey diagram demonstrating the cell–cell interactions of different cell types in 16 organs. The thickness of lines represents the strength of cell–cell interactions. **d** Ligand–receptor interactions between selected cell subtypes in different organs. Each row represents a ligand-receptor pair, and each column defines a pair of cell–cell interaction. P-values were calculated by CellPhoneDB without multiple comparisons.

To decipher the dynamics of intercellular communications in different organs, we employed CellPhoneDB^41^ to identify potential ligand-receptor pairs among the major cell types. We observed that there are strong intercellular interactions among stromal cells, epithelial cells, and myeloid cells (Fig. 5a, b). Generally, the intensity and pattern of intercellular interactions were organ-specific (Fig. 5c). For example, the organs of tongue and uterus showed stronger cellular interactions, while intercellular interactions in testicular were weaker than other organs (Supplementary Figs. 10, 11). To chart the rewiring of molecular interactions regulating cell–cell interactions, we mapped ligand-receptor pairs in specified cell subpopulations in different organs (Fig. 5d). In brief, the “CD99-PILRA” ligand-receptor pair was specific in the interactions between stromal cells and myeloid cells, particularly in adipose, aorta, and colon. As an inhibitory receptor of immunoglobulin-like type 2 receptor (PILR), PILRA has been shown to bind to the CD99 ligand for immune regulation^42^. The “CCL4L2-VSIR” pair occurred exclusively in the interaction of myeloid cells and T cells. In contrast, the “LGALS9-CD44” pair contributed to most immune cell-related interactions. Accordingly, *CD44* plays a role in innate immunity and subsequent adaptive responses, and has extensive inflammatory and proliferative effects on a variety of cell types^43,44^. Taken together, these results reveal the potential molecular mechanisms underlying cell–cell communication in various monkey organs.

### Single-cell chromatin landscape of major organs in cynomolgus monkey

**Fig. 6:**
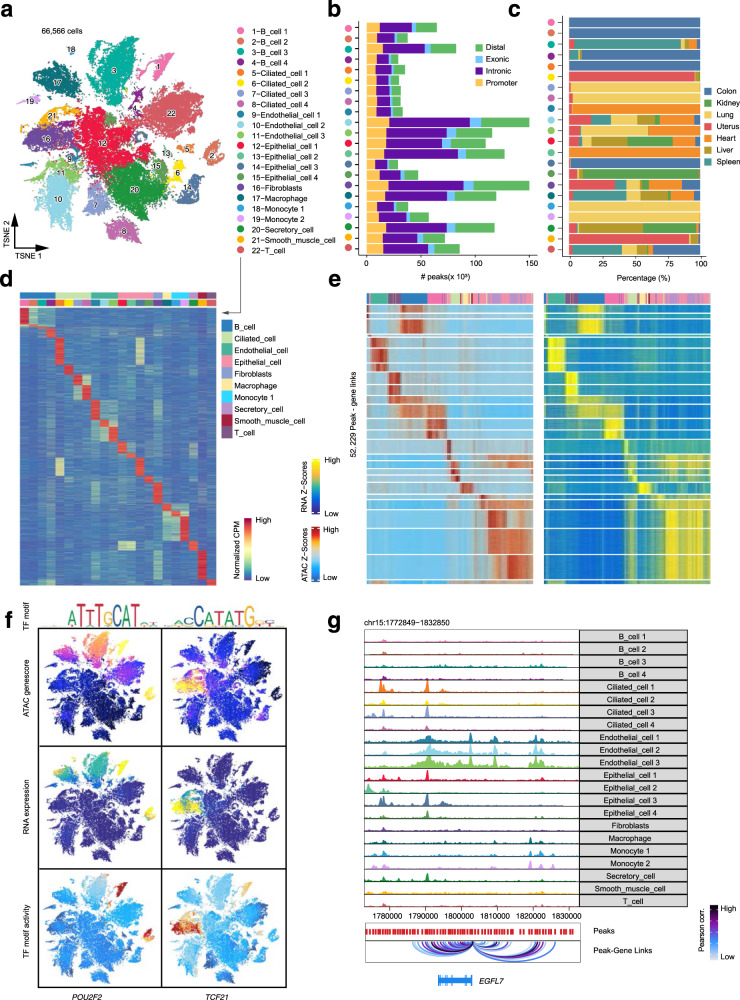
Single-cell chromatin landscape of major organs in cynomolgus monkeys. **a** scATAC-seq data analysis revealing 66,566 cells and 22 cell subtypes. Shown is the t-SNE of cells colored by cell subtypes. **b** Bar plot showing the number of reproducible peaks identified from each cluster. The peaks are classified into four categories: distal, exonic, intronic and promoter. **c** Bar plot dhowing the percentage of cell subtypes in each organ. **d** Heatmap of 80,270 marker peaks across 22 subclusters identified by bias-matched differential testing (FDR < 0.01 and log_2_FC > 3). **e** Heatmap illustrating the chromatin accessibility and gene expression of 52,229 significantly (Pearson correlation *r* > 0.45 and adjusted *p*-value < 0.1, provided by chromVAR) linked peak-gene pairs. **f** Profile of *POU2F2* and *TCF21* chromatin accessibility, gene expression (inferred from scRNA-seq) and TF motif activity. **g** Visualization of the *EGFL7* locus with the maximum number of peak-gene pairs shown by genome browser track (chr15: 1,772,849-1,832,850).

To deconstruct the gene regulation principles of complex tissues in cynomolgus monkey, we examined the single-cell chromatin accessibility landscape of major organs including colon, kidney, lung, uterus, heart, liver, and spleen by scATAC-seq. In total, we generated open chromatin profiles from 66,566 individual cells after quality control. We identified 22 distinct cell clusters in the integrated cell map according to cluster-specific *cis*-elements and visualized the single-cell profiles with uniform manifold approximation and projection (UMAP) (Fig. 6a and Supplementary Fig. 12a). For example, clusters 1-4 demonstrated accessibility at *cis*-elements neighboring B cell genes, such as *CD22*, *MS4A1,* and *TNFRSF13C*, while the cluster 22 exhibited accessibility at *cis*-elements neighboring T cell genes, such as *CD3D* and *IL7R* (Supplementary Fig. 6). We detected 397,773 *cis*-elements across all cell clusters, ranging from 3,046 to 75,001 peaks in each cluster (Fig. 6b). As expected, most of *cis*-regulatory elements (CREs or ATAC peaks) were derived from promoters, intronic or distal intergenic regulatory regions. We observed that most cell clusters were organ-specific (Fig. 6c) and cluster-specific CREs exhibited organ-specific accessibility accordingly (Fig. 6d), suggesting that different cell types and organs have distinctive chromatin landscapes.

Comparison analysis of scATAC-seq and scRNA-seq data highlighted concordant patterns of chromatin accessibility and gene expression across clusters, as exemplified by marker genes (*POU2F2* and *TCF21*) in specific cell types (Fig. 6f). We also computed the transcription factor (TF) deviation score (namely motif activity) using chromVAR^45^, which measured the accessibility of TF binding “footprint” at genome-wide in each single cell. Indeed, the motif activity of *POU2F2*, a B-cell-specific TF involving in cell immune response by regulating B cell proliferation and differentiation^46,47^, was increased in the B cell cluster (Fig. 6f and Supplementary Fig. 12b). Similarly, the motif activity for *TCF21*, an essential regulator of fibroblasts in development^48^, was increased in the fibroblast cell cluster (Fig. 6f and Supplementary Fig. 12b). Furthermore, we applied Cicero^49^ to identify co-accessible *cis*-elements at genome-wide. As exemplified at the gene locus of *EGFL7*, we observed increased enhancer-promoter connections in the endothelial cell cluster at its promoter (Fig. 6g). The result is consistent with the role of *EGFL7* as an endothelium-specific secreted factor mostly produced by blood vessel endothelial cells during development^50–52^.

**Fig. 7:**
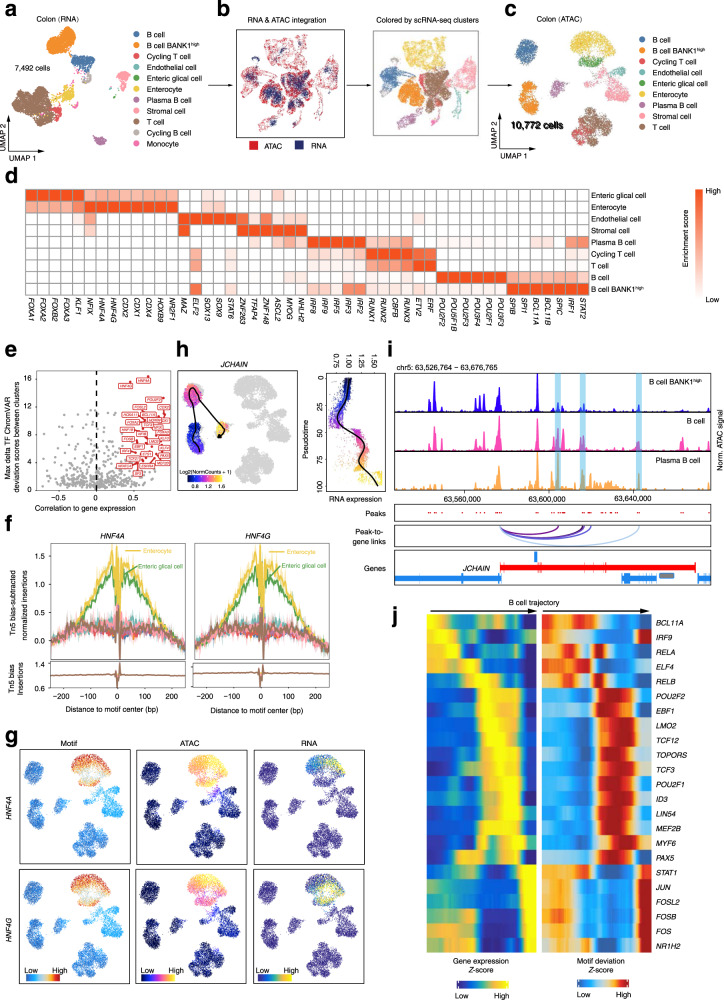
Integrative analysis of scRNA-seq and scATAC-seq data in colon. **a** UMAP plot showing the cell types identified by scRNA-seq data in colon. **b** UMAP plot showing the joint clustering of scRNA-seq (blue) and scATAC-seq (red) data in colon. Cells in the right UMAP are colored based on cell types annotated by scRNA-seq data. **c** UMAP plot showing the colon scATAC-seq cell types, which are inferred by scRNA-seq data. Note that cycling B cell and monocyte are not captured in scATAC-seq assay. **d** The heatmap showing the enrichment of the TF motif in cell-type-specific peaks. **e** Dot plot showing the identification of positive TF regulators through correlation of chromVAR TF deviation scores and gene expression in cell groups (Pearson correlation r >  0.5, adjusted *p*-value < 0.01 and deviation difference in the top 25th percentile). **f** TF footprint for the HNF4A and HNF4G motif with the subtracting the Tn5 bias normalization method. **g** Profile of the *HNF4A* and *HNF4G* gene chromatin accessibility, gene expression (inferred from scRNA-seq), and TF motif activity. **h** The dynamic of chromatin accessibility (left) and gene expression (right) of the *JCHAIN* gene along the B cell pseudo-time trajectory. **i** Genome track visualization of the *JCHAIN* locus (chr5: 63,526,764–63,676,765). Inferred peak-to-gene links for distal regulatory elements are shown below. **j** Heatmap showing the positive TF regulators for which gene expression is positively correlated with TF deviation (inferred by chromVAR) across the B cell trajectory.

To explore the gene regulatory programs in a specific organ, we integrated the gene accessibility and gene expression data in colon using ArchR^33^. Based on the integration, cell type annotations from scRNA-seq were transferred to scATAC-seq using the latent semantic analysis (LSI) (Fig. 7a–c). Therefore, nine cell types were predicted with a high accuracy in scATAC-seq data (Fig. 7c and Supplementary Fig. 13a, b). Two rare cell types, monocyte and cycling B cells, were found in RNA clusters but not in ATAC clusters. This discrepancy might be due to the integration algorithm not sensitive enough to rare cells or cells with close states. We observed that most of ATAC peaks (CREs) demonstrated differential accessibility among cell clusters (Supplementary Fig. 13c), confirming distinctive chromatin landscapes in different cell types. The identities of cell clusters were determined according to the gene-activity score of known cell-specific markers (Fig. 7d). For example, the TF HNF4A is a crucial regulator for enterocyte cell identity^53,54^. Motif analysis revealed that different TF binding motifs showed different degree of enrichment among cell clusters, with the motif enrichment of HNF4A and HNF4G showing the largest variance (Supplementary Fig. 13d, e).

CREs and TFs make up the regulatory logic determining the cell state transition^55,56^. To study CREs for cell type-specific transcriptional programs, we identified a total of 44,156 CRE-gene pairs (peak-to-gene links) in the ATAC-RNA integration cell clusters (Supplementary Fig. 13f). Similar to the observation of cell-specific CREs (Supplementary Fig. 13c), 36% of the identified CRE-gene pairs were also specific to a particular cell type (Supplementary Fig. 14a). Interestingly, we found that CRE-target genes significantly (hypergeometric test, *p*-value < 0.001) overlapped with the differentially expressed genes (DEGs) in each cell type (Supplementary Fig. 14b), supporting the notion that CREs regulate the transcription of target genes. In general, CREs regulated multiple genes, and the number of CREs per target gene was positively corrected with the CRE-gene association especially for local associations (Supplementary Fig. 14c–e). These observations suggest that organ development is subjected to complex regulation via multiple CREs, in line with recent studies^57,58^.

Next, we correlated the motif activity with the expression level of corresponding TFs to systematically deduce either positive or negative TFs controlling colon development based on whether TF expression was positively or negatively correlated with their motif enrichment. Top 30 representative activators were highlighted in Fig. 7e, including cell identities such as *HNF4A*, *HNF4G* and *CDX2* for enterocyte cells (Fig. 7f), *POU2F2* and *BCL11A* for B cells, and pioneer factors *FOXA2*, *FOXA3* and *KLF4*^59^ that play crucial roles in cell fate specification. Generally, the expression of these factors was closely correlated with the accessibility of open chromatin regions and their binding motif enrichment (Fig. 7g and Supplementary Fig. 15).

Furthermore, we sought to demonstrate the power of scATAC-seq data for reconstructing cellular developmental trajectories in colon—the analysis would allow to identify key regulators for organ development at a cell-type level. We focused on the three B cell-related clusters for trajectory analysis since the B-cell developmental trajectory is a well-defined differential programme in other model species^60–63^ that could be used to compare regulatory mechanisms found in monkey. We set the B cell cluster highly expressing *BCL11A* as the start of the trajectory because *BCL11A* has been implicated in early B lymphopoiesis^60,64–66^ (Fig. 7h). We analyzed the dynamics of gene scores and expression, CRE accessibility as well as TF motif enrichment across the differentiation trajectory (Supplementary Fig. 13g). For example, we observed coordinated sequential activation of *JCHAIN* based on both the gene score and gene expression across the trajectory (Fig. 7h). Consistently, the CRE accessibility in the promoter and distal enhancers of *JCHAIN* gradually increased from the early to the intermediate and then to the effector states (Fig. 7i). To identify positive TF regulators driving B-cell differentiation, we integrated motif accessibility with similarly dynamic gene scores or gene expression patterns across the trajectory. We found that *BCL11A*, *IRF9*, *PAX5*, *EBF1*, *POU2F2* and *FOS*, factors that are critical for B cell lineage specification^47,60,67^, showed sequential activities (Fig. 7j).

Overall, our scATAC-seq data provide a rich resource for unbiased discovery of cell types and regulatory DNA elements in cynomolgus monkey.

### Cell-type-specific- and organ-specific transcriptional gene regulatory networks

**Fig. 8:**
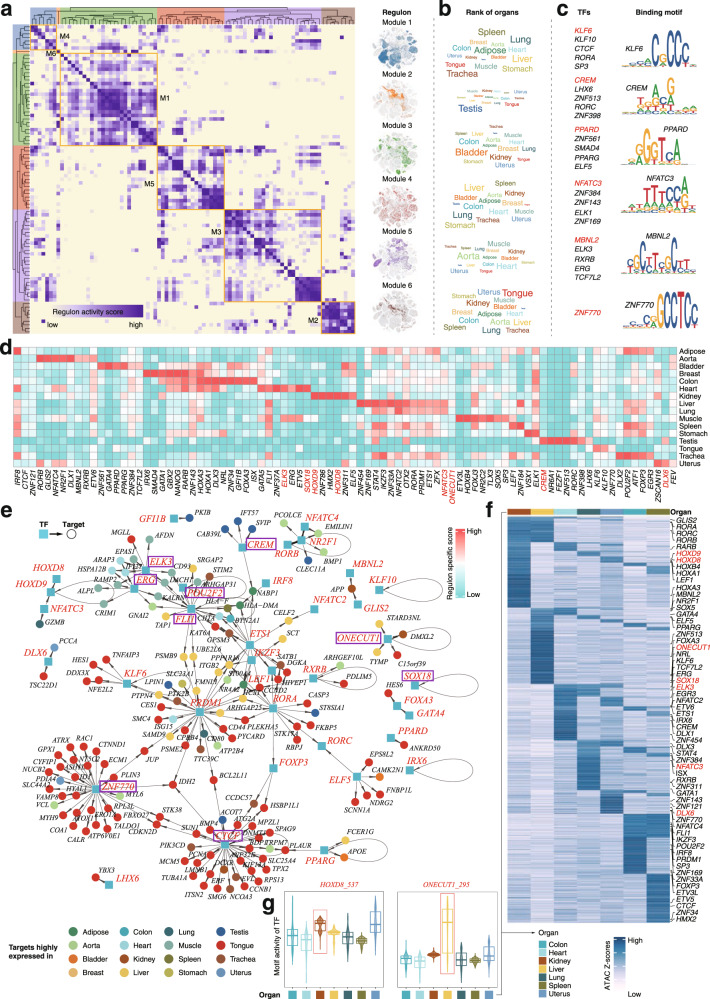
Organ-specific transcriptional regulatory networks. **a** Identification of regulon modules using SCENIC. Heatmap (left) shows the similarity of different regulons (*n* = 87) based on the AUCell score. Eight regulon modules were identified based on regulon similarity. UMAPs (right) illustrate the average AUCell score distribution for different regulon modules (in different colors). **b** Wordcloud plots showing enrichment of organs in different regulon modules. **c** Representative transcription factors (TFs) and corresponding TF binding motifs in different regulon modules. **d** Heatmap showing TFs enriched in different organs. Color depth represents the level of regulon-specific scores. **e** Integrated gene-regulatory networks of the regulons. Regulon-associated TFs are highlighted in blue rectangles and target genes in circles. Target genes (in circles) are colored according to their highly expressed organs. **f** Heatmap showing gene-activity scores of marker genes in the indicated organs. **g** Violin plots showing motif activity (measured by TF chromVAR deviations) of TF regulons highlighted in (**f**). Box plots indicate the median (horizontal line), second to third quartiles (box).

To infer cell-type and organ-specific transcriptional regulatory networks in a systemic manner, we applied SCENIC^4^ to identify TF regulons based on co-expression and motif enrichment. We identified several TF regulon modules that were active in either cell-type (*n* = 8; Supplementary Fig. 16a, b) or organ-specific manners (*n* = 6; Fig. 8a, b). Subsequently, we analyzed representative TF regulons across different cell types (*n* = 7; Supplementary Figs. 16c, 18) or different organs (*n* = 16; Fig. 8c and Supplementary Fig. 17). The identified TF regulons were highly cell-type or organ-specific based on regulon activity scores (Fig. 8d and Supplementary Fig. 16d). Finally, the representative TF regulons and their associated target genes were organized into cell-type-specific or organ-specific gene regulatory networks (Fig. 8e and Supplementary Fig. 16e).

In the cell-type-specific gene regulatory networks, we observed that *CREM* specifically controlled proliferation-related target genes such as *DAZL* and *HEY2* in spermatid cells^68^. Immune-related TFs such as *IRF2*, *FLI1* and *IK2F3* were shown to regulate immune cell identity genes such as *S100A4* and *CD48*^69–71^. *FEV*, a known TF that regulates the development of hematopoietic stem cells^72^, extensively link to target genes actively expressed in immune cells and epithelial cells (Supplementary Fig. 16e).

In the organ-specific gene regulatory networks, we found that target genes of *ZNF770* and *CTCF* were specifically expressed in tongue. The spermatid cell-specific TF regulon CREM regulated genes actively expressed in testis, consistent with its role in spermatid development^68^. ETS-factors (ELK3, ERG, and FLI1) together with pre-/immature-B TFs (POU2F2) positively regulated genes showed elevated activity in heart and muscle^73,74^ (Fig. 8e).

To further confirm the unbiased inference of organ-specific TF regulons based on scRNA-seq data (Fig. 8d), we validated the organ-specific TF regulons using organ-matched scATAC-seq data. Chromatin accessibility were measured at *cis*-elements containing a specific TF binding motif using chromVAR^45^ and accessibility changes were analyzed in binding sites for the above-identified organ-specific TFs (denoted as TF deviation scores). In general, TF deviation scores showed similar organ-specific patterns to regulon activity scores (Fig. 8f). For example, the HOXD8 regulon was kidney-specific and the TF deviation scores of HOXD8 showed relatively high levels in kidney (Fig. 8g). Interestingly, the CRE accessibility and the expression level of the gene *HOXD8* itself exclusively increased in kidney (Supplementary Fig. 19a, b). These observations indicate that *HOXD8* may be important for regulating the kidney function. Supporting this line of notion, the defection of *HOXD8* is reminiscent of polycystic kidney disease in mouse^75^. We also observed that both the regulon activity and the TF deviation scores of ONECUT1 were liver-specific (Fig. 8g). *ONECUT1* has been shown to play an important role in liver development and was tightly linked to hepatic TF networks that include FOXA3^76–78^. These analyses emphasize the unbiased prediction of organ-specific gene regulatory networks at the single-cell level.

### Comparison of cell landscapes among human, mouse and cynomolgus monkey

**Fig. 9:**
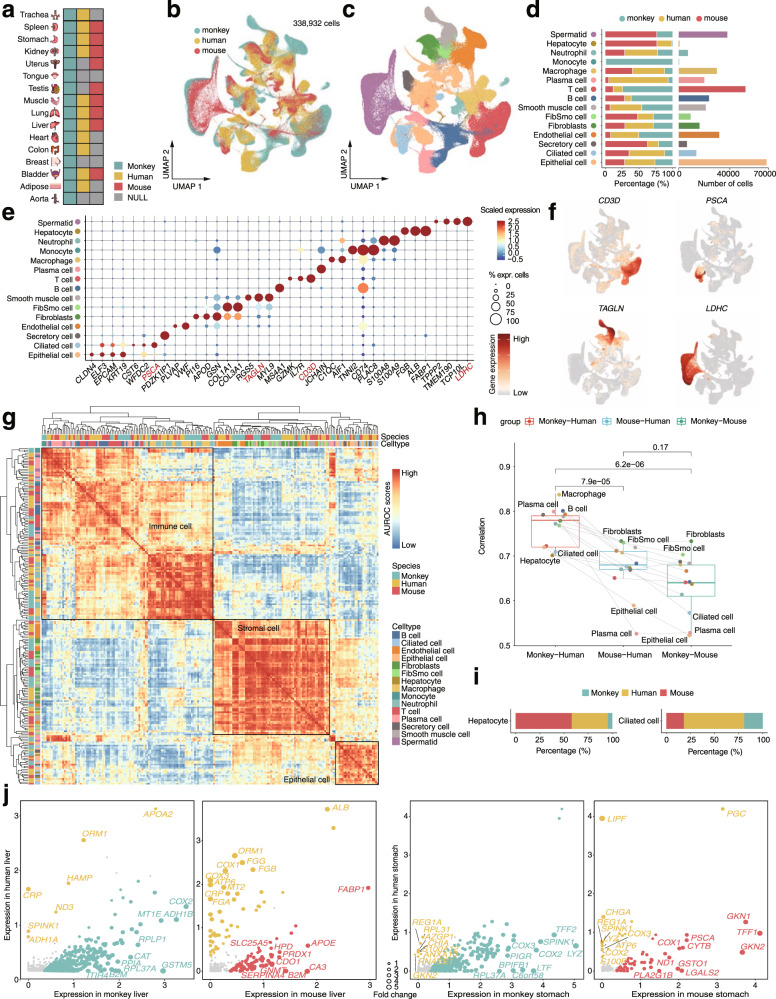
Comparison of single-cell landscapes in human, mouse and cynomolgus monkey. **a** Integration of scRNA-seq data for 16 organs from cynomolgus monkey, eleven organs from human, and nine organs from mouse. **b** UMAP showing the distribution of cells from cynomolgus monkey, human and mouse. **c** UMAP illustrating the distribution of annotated 15 major cell types. **d** Bar plot showing the percentage of cells from the three species (left) and the number of cells in each cell type (right). **e** Dot plot showing representative marker genes of different cell types. Dot size is proportional to the fraction of cells expressing specific genes. Color intensity corresponds to the relative expression of specific genes. **f** Feature Plot showing the expression of selected marker genes. **g** Correlation of orthologous gene expression between human, mouse and monkey pseudo-cell types (*n* = 212) based on the AUROC scores. The AUROC scores were calculated by MetaNeighbor to measure the similarity of different cell types. The clustered heatmap was plotted using the ‘pheatmap’ function in R, where the complete linkage method was used for hierarchical clustering. **h** Box plots showing the Spearman correlation of average gene expression between two different species using top 20 marker genes in a specific cell type. Each dot represents a major cell type (*n* = 13). Statistical significance of difference between comparisons was calculated by the two-sided Mann–Whitney *U* test. The boxes indicate the 25% quantile, median (horizontal line), 75% quantile. **i** The proportion of hepatocytes in liver (left) and ciliated cells in stomach (right). **j** Scatter plots showing a pairwise comparison of gene expression across species in a specific organ. Left two scatter plots are for comparisons of hepatocyte cells in liver and right for comparisons of ciliated cells in stomach. Differentially expressed genes (DEGs) are highlighted and representative DEGs are labeled. The size of dots is proportional to the fold change for a specific gene.

The cynomolgus monkey cell landscape offers the opportunity to compare the cellular components and transcriptomic dynamics across species with similar organ compositions. Here we integrated scRNA-seq data from the non-human primate cynomolgus monkey (by our study), human^4^ and mouse^6^ with matched organs/tissues using orthologous genes for cross-species analysis (Fig. 9a and Supplementary Fig. 20a, b). The integrated cell map consists of 338,932 cells (Fig. 9b), which were grouped into 15 major cell types (Fig. 9c). While the proportions of non-immune cell types were generally stable in the three species, the compositions of certain types of immune cells largely varied among the species (Fig. 9d). In particular, monocytes were rare cells but exclusively detected in monkey. We could rule out the potential bias due to cross-species integration analysis by checking the cell-type annotation in the original cell maps (without integration) of each species (Supplementary Fig. 21 and Supplementary Fig. 22). Instead, the discrepancy might be related to a bias using different platforms to capture rare cell types or selecting sample parts for sequencing. Nevertheless, the expression patterns of representative marker genes and transcriptomic similarity of cell types were overall consistent across species (Fig. 9e, f and Supplementary Fig. 20c, d). Indeed, the gene expression patterns of the major cell types were conserved in all three species, including immune, stromal and epithelial cells (Fig. 9g). This observation is consistent with previous single-cell comparative analyses^4,16–18^. As expected, monkey and human showed significantly higher (Mann–Whitney test, *p*-value < 0.001) cell-type similarity in terms of orthologous gene expression than other comparisons (Fig. 9h). Intriguingly, immune-related cell types (such as macrophages, B cells and plasma cells) showed higher similarities of gene expression between human and monkey than non-immune cells (such as ciliated and epithelial cells). In contrast, stromal cells (including fibroblasts and FibSmo cells) demonstrated the highest similarities in human-mouse and monkey-mouse comparisons (Fig. 9h and Supplementary Fig. 20g). These findings indicate that monkeys share overall highly similar transcriptional programs in the immune system with human, and thus may provide an ideal benchmark for investigating the immune response to diseases such as cancer^79^ and the current coronavirus disease 2019 (COVID-19)^80–86^.

Next, we investigated the transcriptomic dynamics of the same cell types between monkey and other species, with a specific focus on varying cell types in compositions across species. Particularly, hepatocytes and ciliated cells showed the lowest similarity between human and monkey (Fig. 9h and Supplementary Fig. 20c, d) and were mainly enriched in the monkey organs of liver and stomach, respectively (Supplementary Fig. 20e, f). However, compared to human and mouse, both cell types were underrepresented in monkey (Fig. 9i). Therefore, hepatocytes and ciliated cells were two representative cell types that largely different between monkey and human or mouse. We performed pairwise comparison of gene expression in liver for hepatocytes and in stomach for ciliated cells (Fig. 9j). In the differential analysis of gastric ciliary cells between human and monkey, we found that *LYZ* was highly expressed in monkey^87^. As *LYZ* has a dual role of immune defense and digestive function^88^, this observation may highlight that monkey has enhanced function of immune response and digestive system. As a gastric lipase, *LIPF* is expressed in human chief cells and promotes lipid metabolism^89^ and it may help for the fat digestion processes occurring in human. *ORM1*, as an acute-phase protein, was highly specifically expressed in human hepatocytes and had a certain promoting effect on liver regeneration^90^, which might provide a potentially therapeutic target in hepatopathy.

**Fig. 10:**
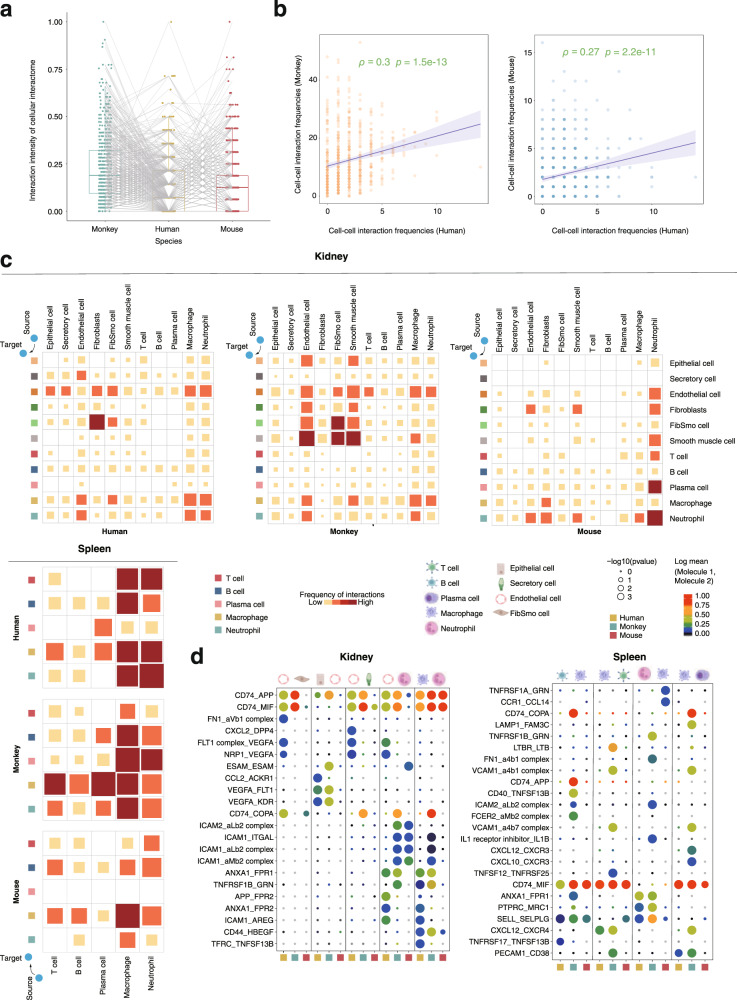
Conserved and divergent cell–cell interactions in human, mouse and cynomolgus monkey. **a** Box plot indicating the relative frequency of cell–cell interactions among human, monkey and mouse. Each point denotes a specific intercellular interaction in a specific organ. The number of significant cell–cell interactions are scaled to a range from 0 and 1 for inter-species comparison. **b** Scatter plots show the Spearman’s rank correlation of cell–cell interaction frequencies between human and monkey (left) or between human and mouse (right). *P*-values are provided (two-sided Spearman’s correlation test). The fitted line and standard errors with 95% confidence intervals are shown. **c** The heatmaps showing the strength of interactions among the common major cell types in kidney and spleen in the three species. The size and color of the blocks are proportional to the frequency of interactions. *P*-values were calculated by CellPhoneDB without multiple comparisons. **d** Dot plot of interactions between major cell subtypes in kidneys and spleens of different species. Each row represents a ligand-receptor pair, and each column defines a cell–cell interaction pair in a species.

Finally, we explored conserved and divergent patterns of cell–cell communications among the three species. We performed intercellular ligand-receptor interaction analyses between each pair of cell types in each organ using CellPhoneDB^41^. The number of significant cell–cell interactions were counted in each species and then scaled to the range between 0 and 1 for inter-species comparison (Fig. 10a). Interestingly, the frequency of intercellular interactions was positively correlated between different species (Fig. 10b), suggesting that these species generally share common intercellular signaling pathways mediating cell–cell communications. The overall intercellular interaction pattern was more similar, albeit slightly, between human and monkey than that between human and mouse (Spearman correlations 0.30 versus 0.27; Fig. 10b). Indeed, the interaction intensities of immune-related cell–cell interactome and the corresponding top ligand-receptor pairs were more consistent between human and monkey in the investigated organs such as kidney and spleen (Fig. 10c, d). In contrast, intercellular interactions among non-immune cells preferred to be more consistent between monkey and mouse (Supplementary Fig. 23).

## Discussion

Non-human primates (NHP) are similar to humans in terms of anatomy, physiology and biochemical metabolism. Cynomolgus monkeys, a well-established laboratory animal model, have outstanding contributions to the scientific field^91^. Although several single-cell transcriptomic atlases have recently been established in cynomolgus monkeys based on a few organs (including ovary, lung, heart and artery)^28–30^, an organism-wide single-cell map is still lacking in this model species. Here, we charted a reference cell map of cynomolgus monkeys (named ‘Monkey Atlas’) using both scATAC-seq and scRNA-seq data across multiple organs, allowing deeper insights into the molecular dynamics and cellular heterogeneity of the cynomolgus monkeys organism.

As a proof of concept, we have performed various analyses based on the Monkey Atlas to show its wide uses, including the discovery of new putative cell types, the identification of key regulators in organ specification, and the ability to compare cell types across organs and species. For instance, our data demonstrated that ciliated cells present in various organs of cynomolgus monkeys and the different ciliated cell subpopulations showed various functions related to metabolic process, signal transduction, and cellular response to stimulus (Fig. 2). This observation somehow expands our notion that ciliated cells are generally found in respiratory system^92^ with vital role in cleaning pathogenic microorganisms and signal transduction^36^. We also predicted key regulatory factors controlling gene expression patterns of different cell types. Consistent with previous studies^93–95^, SPIB, POU2F2, SPI1, CEBPD and IRF4 were key regulators in myeloid cells and the motifs of these TFs were overrepresented in Monocytes_IL1B^high^ cells than other cells in the SCENIC analysis. In addition to the discovery of known immune-related TFs regulating S100A4 and CD48, we also found FEV, a known TF that regulates the function of hematopoietic stem cells^72^, has shown extensive regulation of other TFs in immune cells and epithelial cells in our study.

Recently, comprehensive reference cell maps across organs have been established in human^4,5^ and mouse^6,96^. Although the Monkey Atlas did not provide exhaustive characterization of all organs in cynomolgus monkeys, it does offer a rich dataset of the most populously studied organs in biology. In this regard, we performed cross-species integration analysis of cell maps to explore the molecular and cellular differences among the three species (human, cynomolgus monkeys and mouse) with comprehensive single-cell data. We noticed that cynomolgus monkeys and human both have abundant immune cells and epithelial cells and a comparative composition of cell types in matched organs. This indicates that cynomolgus monkeys are ideal models to study complex diseases. The differences in cell compositions and gene expression of human, mouse and monkey cells may provide scientists with a guide basis for selecting experimental animals.

Although we have provided relatively rich single-cell multi-omics data in cynomolgus monkey, there are still several limitations of the current study. First, only one male and one female monkeys were included in the analysis. It is therefore challenging to explore genes with sex-biased expression and other sex-related differences in our analysis. Second, the majority of the samples were taken from a single individual monkey. We cannot assess the impact of substantial cellular heterogeneity within the same organ or tissue. Nevertheless, there is a good match of expression patterns and cell compositions between male and female monkeys in the organs of liver, heart and colon. Third, there are still many functionally important organs or tissues (such as ovary, pancreas, and cerebellum) that have not yet been included in our study due to limited resources. In addition, some scRNA-seq samples do not have matched scATAC-seq data, which may restrict unbiased exploration of DNA regulatory elements in specific organs.

During the revision of our manuscript, we acknowledge that a very recent study by Han et al. provided a large-scale cell transcriptomic atlas in cynomolgus monkeys^97^. Both this study and ours bear striking similarities in terms of investigated organs/tissues and annotated cell components. Regardless of the differences of analytical focuses between the two studies, there are at least three aspects to demonstrate that the two studies are complementary to each other. First, although Han et al. included more tissue samples (n = 45), there are some organs / tissues (including breast and muscle) not covered but included in our data. Second, Han et al. mainly adopted single-nucleus RNA sequencing (snRNA-seq) to profile frozen tissues. However, nuclei have lower amounts of mRNA and less complexity of cell types compared to cells^98^. In contrast, our scRNA-seq data generated from fresh tissues provide more information including both cytoplasmic and nuclear transcripts, allowing to annotate potential new but rare cell types (such as neutrophils and spermatid cells) which were not mentioned by Han et al.^98^. Lastly, Han et al. provides only one scATAC-seq sample in kidney, while the eight samples in seven different organs provided in our study is a well complement to the cynomolgus monkey database.

In conclusion, our Monkey Atlas together with the cell transcriptomic atlas by Han et al.^98^ provide valuable information about the most populous and important cell populations in cynomolgus monkey, which are stepping stones for preclinical studies in future.

## Methods

### Organ tissue collection

Two healthy four-year-old cynomolgus monkeys were raised from the monkey breeding base of Changchun Biotechnology Development Co., Ltd., Guangxi, China. The managing protocols of the monkeys were carried out in accordance with the standard procedures referring to Guide for Care and Use of Laboratory Animal (2010) and the principles on Animal Welfare Management (Public Law 99-198). The sample collection of cynomolgus monkey and research conduction in this study were approved by the Research Ethics Committee of the Changchun Biotechnology Development Co., Ltd. (Approval Number: 21001). Tissues were collected from 16 different organs, including trachea, spleen, stomach, kidney, uterus, tongue, testis, muscle, lung, liver, heart, colon, breast, bladder, adipose and aorta. Specifically, tissues were firstly cut into 1-2 mm^3^ pieces in RPMI-1640 medium (Gibico) with 12% fetal bovine serum (FBS, Gibico), then enzymatically digested with gentleMACS (Miltenyi) according to manufacturer’s instruction. Cells were passed through a 70 μm cell trainer (Miltenyi) and centrifuged at 300 g for 5 min at 4 °C. The pelleted cells were re-suspended in red blood cell lysisbuffer (Beyotime) and incubated 1 min to lyse red blood cells. After wash twice with 1XPBS (Gibico), the cell pellets were re-suspended in sorting buffer (PBS with supplemented with 1% FBS). Single cells were captured by the 10 × Genomics Chromium Single Cell 3’ Solution. scRNA-seq library was conducted by Shanghai Xuran Biotechnology and scATAC-seq library was conducted by LC-Biotechnology (Hangzhou, China) and prepared following the manufacturer’s protocol (10 × Genomics). The libraries were subjected to high-throughput sequencing on the Novaseq6000 platform, and 150-bp paired-end reads were generated.

### scRNA-seq and data processing

The reference genome sequence of *Macaca fascicularis* in FASTA format and gene annotation in GTF format were downloaded from the ENSEMBL database. Raw scRNA-seq data were aligned to the *M.fascicularis* reference genome (macFas6), and subjected to barcode assignment and unique molecular identifier (UMI) counting using the CellRanger v3.1.0 pipeline (10x Genomics). Filtered count matrices from the CellRanger pipeline were converted to sparse matrices using Seurat package (v4.0.0)^99^. Potential doublets were detected and filtered using DoubletFinder^100^ based on the expression proximity of each cell to artificial doublets. Cells which expressed either more than 4000 genes or less than 200 genes, as well as the ones who has more than 20% of mitochondrial gene expression in UMI counts were removed from the analysis. Filtered data were then log normalized and scaled to avoid cell-to-cell variation caused by UMI counts and the percent mitochondrial reads. Specifically, the top 3000 most variably expressed genes were determined using the “vst” method in the “FindVariableFeatures” function and scaled using “ScaleData” with regression on the proportion of mitochondrial UMIs (mt.percent).

We used the Robust Principal Component Analysis (RPCA) method in Seurat for integration of scRNA-seq data from different organs. The “RunPCA” function was used to compute the top 20 principal components (PCs) using variably expressed genes. We used UMAP (Uniform Manifold Approximation and Projection) for visualization of cell clusters. Clustering was performed for integrated expression values based on shared-nearest-neighbor (SNN) graph clustering (Louvain community detection-based method) using “FindClusters” with a resolution of 0.8. We used the “FindAllMarkers” function with default parameters to identify markers for each cluster. Marker genes for each cluster were provided in Supplementary Table 2.

### Pathway analysis

Gene-set enrichment analysis on differentially expressed genes (DEGs) in this study was performed by the clusterProfiler package^101^ in R. Gene-set variation analysis (GSVA) was conducted using the GSVA^102^ package. Expression differences between different cell groups were calculated by the ‘FindMarkers’ function in the Seurat package. UCell^103^ was used to calculate the gene signature scores of the collagen metabolic pathway, which includes 24 genes (*CST3, CTSK, CTSS, FAP, MMP14, MMP16, MMP9, VSIR, ADAMTS3, COL1A2, CREB3L1, F2, F2R, HIF1A, IL6, LARP6, P3H1, RGCC, SERPINF2, SERPINH1, SMPD3, TNS2, TRAM2,* and *VIM*) annotated in the genome of *M.fascicularis*.

### Trajectory inference using Monocle

Monocle2 (version 2.99.3)^34^ was used to infer the epithelial cells state transition. The UMI count matrix of epithelial cells, gene and cell annotation information derived from Seurat analysis were used to create a CellDataSet object. Variable genes identified among epithelial cell subsets were used to sort cells in the pseudotime analysis. We used the DDRTree method and orderCells function for dimensional reduction and cell ordering. The ciliated_cell_SCGB1D2^high^ (E09 for the bladder organ) or ciliated_cell_PTGR1^high^ clusters (E12 for other organs) were defined as the root state of the inferred trajectory.

### RNA velocity analysis

The generated bam files by CellRanger were sorted by SAMTools. The sorted bam files were then used to run the ‘run10x’ command from Velocyto to generate a loom file. RNA velocity analysis was independently performed in epithelial cells and stromal cells.

### Cell–cell interaction analysis

Cell–cell interactions among different cell types were estimated by CellPhoneDB (v2.1.1)^41^ with default parameters (10% of cells expressing the ligand/receptor). In order to run CellPhoneDB analysis in cynomolgus monkeys, the *M.fascicularis* genes were converted to human genes based on homologous gene mapping. Interactions with *p*-value < 0.05 were considered to be significant. We considered only ligand-receptor interactions based on the annotation from the CellPhoneDB database, and discarded receptor-receptor and other interactions without a clear receptor.

### Create a cisTarget database for *Macaca fascicularis*

We followed the instruction by SCENIC (https://github.com/aertslab/create_cisTarget_databases) to construct cisTarget database. Since there are no well annotated TF motifs in *M.fascicularis*, we instead used the annotated human motifs from the CIS-BP website (http://cisbp.ccbr.utoronto.ca/) to create cisTarget databases for *M.fascicularis*.

### Gene-regulatory network

To identify cell-type and organ-specific gene regulatory networks, we performed Single-cell Regulatory Network Inference and Clustering (v0.10.0; a Python implementation of SCENIC)^3^ in our *M.fascicularis* dataset. Firstly, the original expression data were normalized by dividing the gene count for each cell by the total number of cells in that cell and multiplying by 10,000, followed by a log1p transformation. Next, normalized counts were used to generate the co-expression module with GRNboost2 algorithm implemented in the arboreto package (v.0.1.3). Finally, we used pySCENIC with its default parameters to infer co-expression modules using the above-created RcisTarget database. An AUCell value matrix was generated to represent the activity of regulators in each cell. The final cell-type and organ-specific gene regulatory networks (GRNs) consisted of 86 and 87 regulons for our *M.fascicularis* dataset as shown in Supplementary Fig. 16a and Fig. 8a. GRNs were visualized by the igraph package in R.

### Cross-species analysis of multiple-organ scRNA-seq data

For cross-species comparison analysis of scRNA-seq data, we only included the one-by-one orthologous genes (n = 12,971) for subsequent analysis. Specifically, cell count matrices of the orthologous genes were extracted from the integrated scRNA-seq data from cynomolgus monkey, human and mouse, respectively. Only cells in matched organs/tissues were considered in the analysis. We then performed cross-species scRNA-seq data integration using the Seurat’s reciprocal PCA (RPCA) integration strategy. Downstream cell-cluster-based cell-type annotation and marker gene analyses were carried out in a similar way as described above.

To compare the cell-type similarity among the three species, we computed the Spearman’s rank correlation of average expression values of the top 20 marker genes between different species in a specific cell type (Fig. 9h). We also validated the correlation analysis by using different sets of top marker genes (Supplementary Fig. 20g).

To compare the pattern of cell–cell communications among the three species, potential intercellular ligand-receptor interactions between each pair of cell types in each organ of each species were predicted by CellPhoneDB. The number of significant cell–cell interactions (*p*-value < 0.05) were counted in each species for inter-species comparison.

### scATAC-seq data pre-processing

The scATAC-seq sequencing data were pre-processed by cellranger-atac (v1.2.0). The running parameters were used by default except for “--force-cells”. The “--force-cell” was set as 10000 for liver, lung and colon, 8000 for spleen, and default for the rest organs. Subsequent scATAC-seq data analysis was performed by ArchR (v1.0.1)^33^. Specifically, the *M.fascicularis* genome was constructed and annotated by createGenomeAnnotation and createGeneAnnotation function respectively. Then arrow file was created by createArrowFiles function with default parameters. We used the addDoubletScores function to infer potential doublets, and the filterDoublets function was used to remove the potential doublets with the “filterRatio = 1.0” parameter. ArchR project was created by ArchRProject function with default parameters. For dimensionality reduction, we used the addIterativeLSI function in ArchR with the following parameters: “iterations = 4, clusterParams = list (resolution = c(0.2, 0.4, 0.6), sampleCells = 10,000, n.start = 10, maxClusters = 6), varFeatures = 20,000, dimsToUse = 1:50, scaleDims = FALSE”. Next, the Harmony method was utilized to remove the batch effect by the addHarmony function^32^. AddClusters function was used to cluster cells by its default parameters. For single-cell embedding, we selected the reducedDims object with harmony and used addTSNE function with the parameter “perplexity = 30” for visualization.

### Marker genes identification and cluster annotation

To identify the marker gene, gene scores were calculated when the ArchR project was created and stored in the arrow file. Then getMarkerFeatures function was used to identify the cluster-specific “expressed” genes with default parameters. To visualize the marker genes in the embedding, we used addImputeWeights function to run the MAGIC^104^ to smooth gene scores across the nearby cells. For track plot, we used the plotBrowserTrack function with default parameters except for “tileSize = 100”.

### Peak calling and TF binding motif analysis

Before peak calling, we used the addGroupCoverages function with default parameters to make pseudo-bulk replicates. Then the addReproduciblePeakSet function was used with its default parameters except for “genomeSize = 2.7e09” to call accessible chromatin peaks using MACS2(v2.2.7.1)^105^. For cell type-specific peak analysis, the getMarkerFeatures function was firstly applied to identify marker peaks. Then the getMarkers function with the parameter “cutOff = FDR <  = 0.01 & Log2FC >  = 1” was conducted to get the differential peaks. Motif annotation was added to the ArchR project by the addMotifAnnotations function. The TF motif enrichment in differential peaks was computed by the peakAnnoEnrichment function with the parameter “cutOff = FDR <  = 0.1 & Log2FC >  = 0.5”. For motif footprint analysis, we first used the getPositions function to locate relevant motifs; then the getFootprints function was used to compute interested motif footprints with its default parameters. Lastly, footprint patterns were illustrated by the plotFootprints function with the parameter of “normMethod = Subtract, smoothWindow = 10”.

### Integrative analysis of scRNA-seq and scATAC-seq data

In order to align and integrate scATAC-seq data from different organs, we extracted and annotated the scRNA-seq data with matched scATAC-seq data in the same organ. We first used the FindTransferAnchors function from the Seurat package and aligned the data with the addGeneIntegrationMatrix function in ArchR with “unconstrained integration” mode. Most of the predicted scores were > 0.5 in the result, indicting a relatively high prediction accuracy. To improve the accuracy of the prediction and to better integrate the two datasets, a “constrained integration” mode was applied to integrate the scATAC-seq and scRNA-seq data. Briefly, we annotated the scATAC-seq data with cell types based on the gene scores of scATAC-seq. Then, a restricted list was created to make sure that gene expression similarity was calculated only in the same cell type for both scATAC-seq and scRNA-seq data. For peak-to-gene linkage analysis, we used the addPeak2GeneLinks function to compute peak accessibility and gene expression with the parameters of “corCutOff = 0.45, resolution = 1”.

### Statistical and reproducibility

If not specified, all statistical analyses and data visualization were done in R (version 4.0.0). We state that no statistical method was used to predetermine sample size. No data were excluded from the analyses and the experiments were not randomized. The Investigators were not blinded to allocation during experiments and outcome assessment.

### Reporting summary

Further information on research design is available in the Nature Research Reporting Summary linked to this article.

## Supplementary information

Supplementary Information

Reporting Summary

## Data Availability

The data that support the findings of this study have been deposited into CNGB Sequence Archive (CNSA) of China National GeneBank DataBase (CNGBdb) with accession numbers “CNP0002427” for scRNA-seq data and “CNP0002441” for scATAC-seq data. Gene counts and metadata are available at “Zenodo [10.5281/zenodo.5881495]”. The Gene Expression Omnibus (GEO) accession number for scRNA-seq is “GSE196792”. The GEO accession number for scATAC-seq is “GSE196791”. We also provided an interactive website [https://biobigdata.nju.edu.cn/MonkeyAtlas/] for exploration of marker gene expression based on scRNA-seq data. The public dataset used in this study for cross-species comparisons between humans, mice, and monkeys can be accessed as below: the human count matrix is available at “human count matrix [https://figshare.com/articles/HCL_DGE_Data/7235471]”; the mouse count matrix is available at “mouse count matrix [https://figshare.com/articles/MCA_DGE_Data/5435866]”. All other relevant data supporting the key findings of this study are available within the article and its Supplementary Information files or from the corresponding author upon reasonable request.
