# Supplementary material for: A reference single-cell regulomic and transcriptomic map of cynomolgus monkeys
Source: Nat Commun. 2022 Jul 13;13:4069. doi: 10.1038/s41467-022-31770-x (PMC9279386; doi:10.1038/s41467-022-31770-x)
Supplement: Supplementary file 1 — Supplementary Information [file 41467_2022_31770_MOESM1_ESM.pdf]

Supplementary Information for "A reference single-cell regulomic and transcriptomic map of cynomolgus monkeys"

by Qu et al.

Supplementary Figures

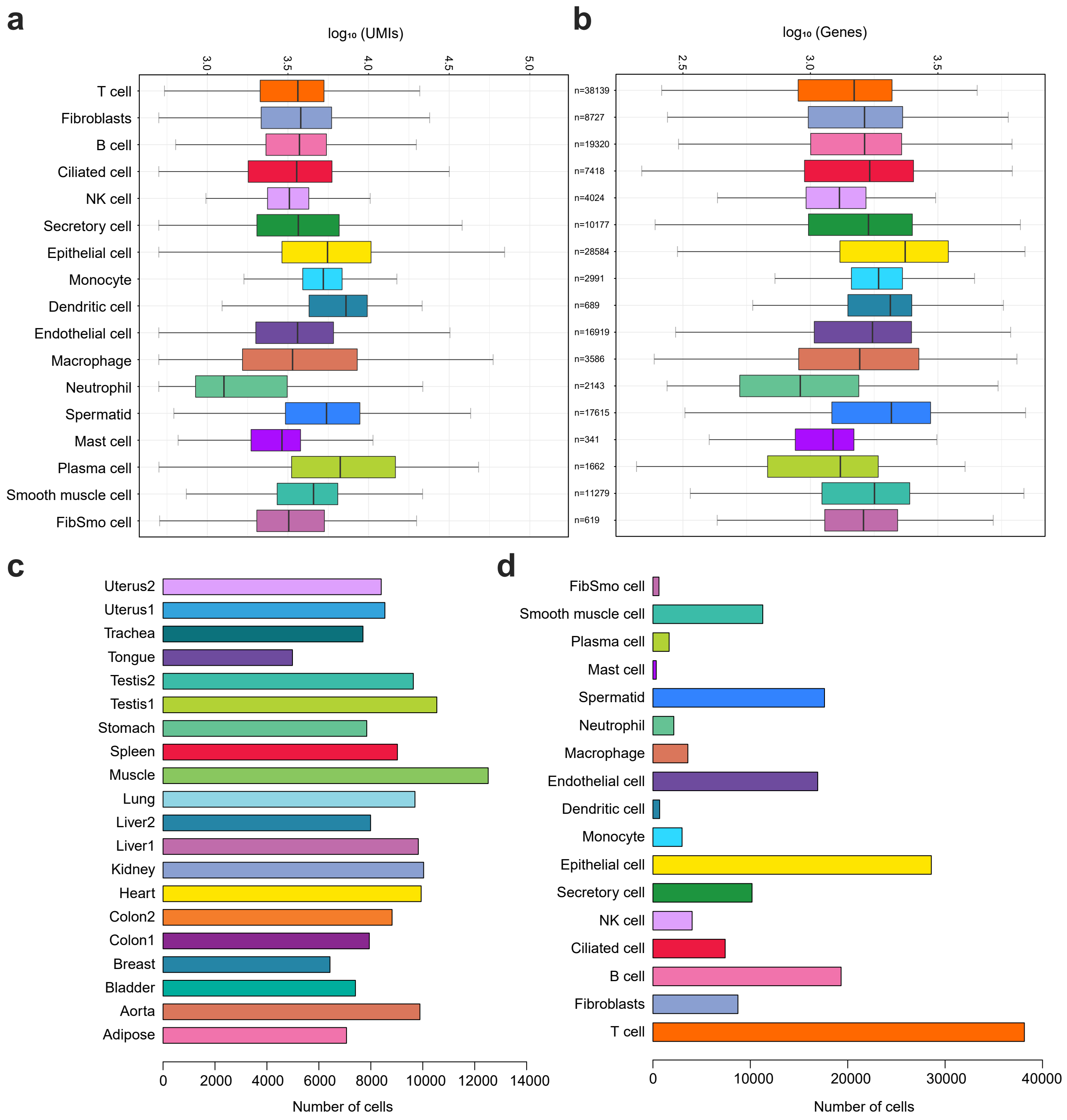

Supplementary Fig. 1. Quality control of scRNA-seq data. Related to Figure 1. **(a-b)** Box plot showing the number of UMIs and genes in major cell types respectively. The boxes indicate the 25% quantile, median (horizontal line), 75% quantile. **(c-d)** Bar plots showing the number of cells in major cell types and in different samples.

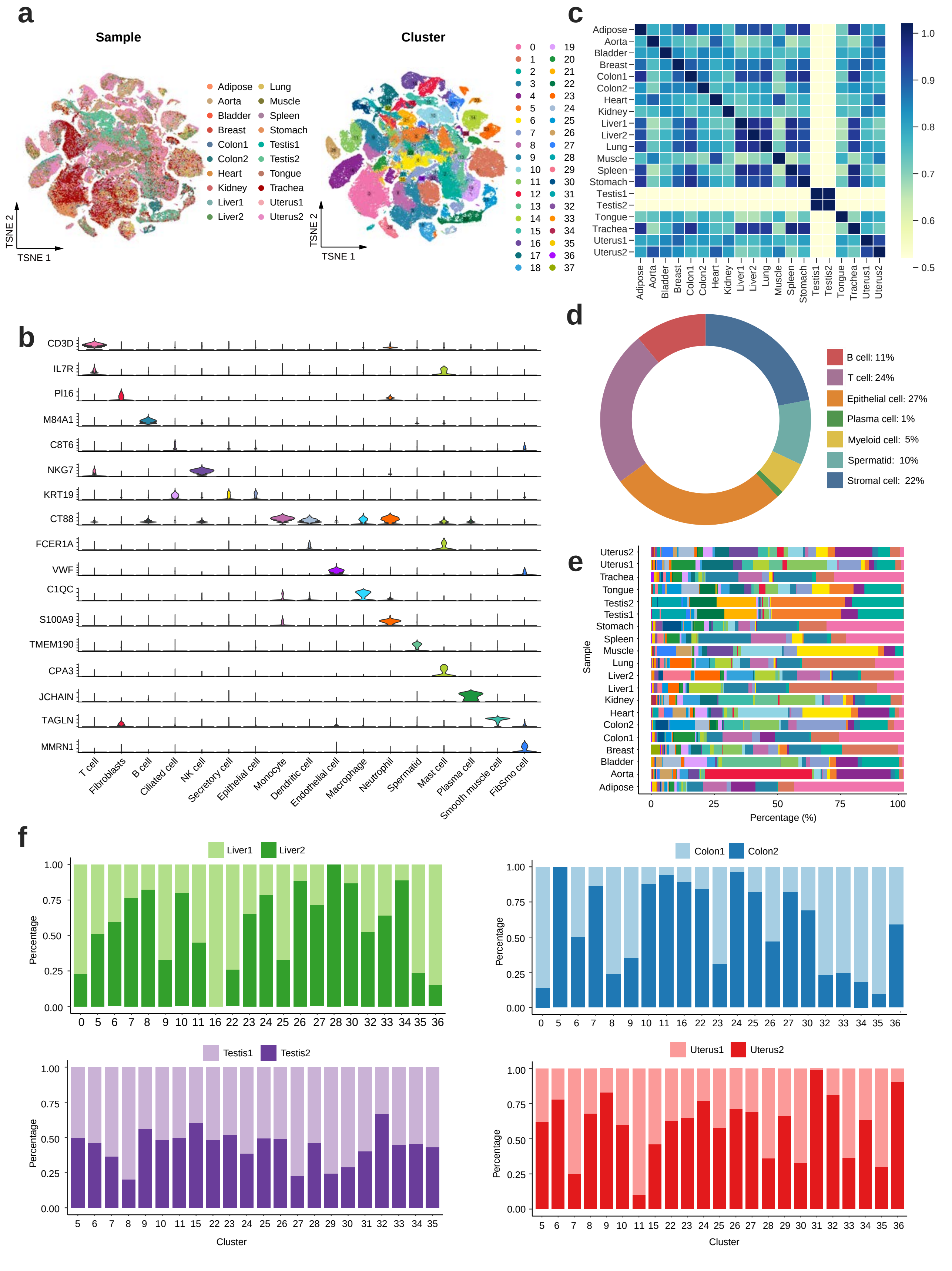

Supplementary Fig. 2. Cell type annotation based on scRNA-seq data. Related to Figure 1. **(a)** t-SNE plot showing the cell distribution according to samples (left) or clusters (right). **(b)** Violin diagram showing the expression of marker genes in major cell types. **(c)** Heatmap showing the expression correlation of samples based on highly expressed genes. **(d)** Circle diagram showing the proportion of cells across the seven major cell types. **(e)** Bar plot showing the percentage of cells in each sample. **(f)** Bar plot showing the proportion of cells in different clusters for organs with replicate samples, including liver, colon, testis and uterus.

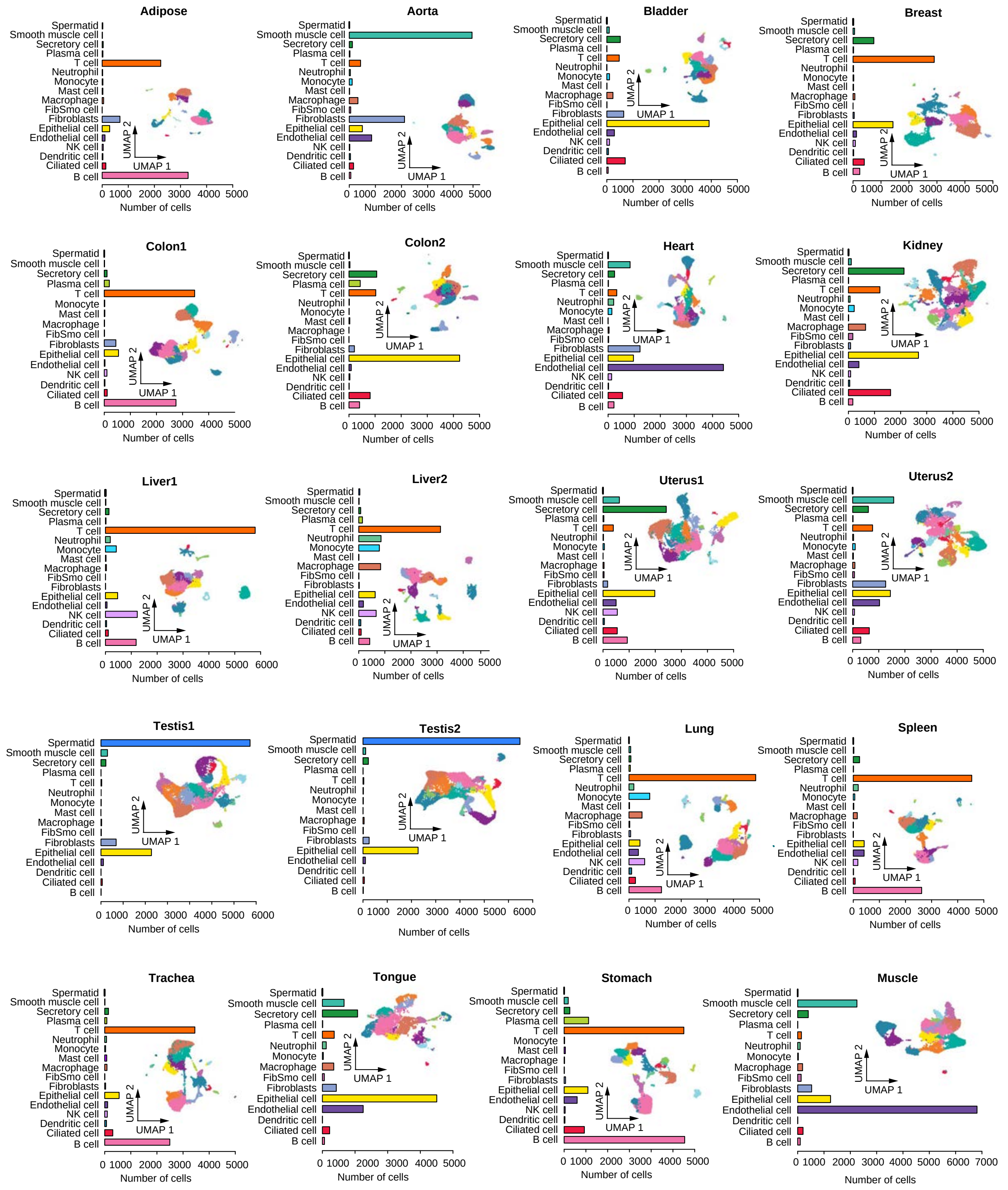

Supplementary Fig. 3. Cell clustering based on UMAP and the proportion of cell types in each organ. Related to Figure 1. UMAPs showing the distribution of cell clusters, and bar plots showing the proportion of cells across major cell types.

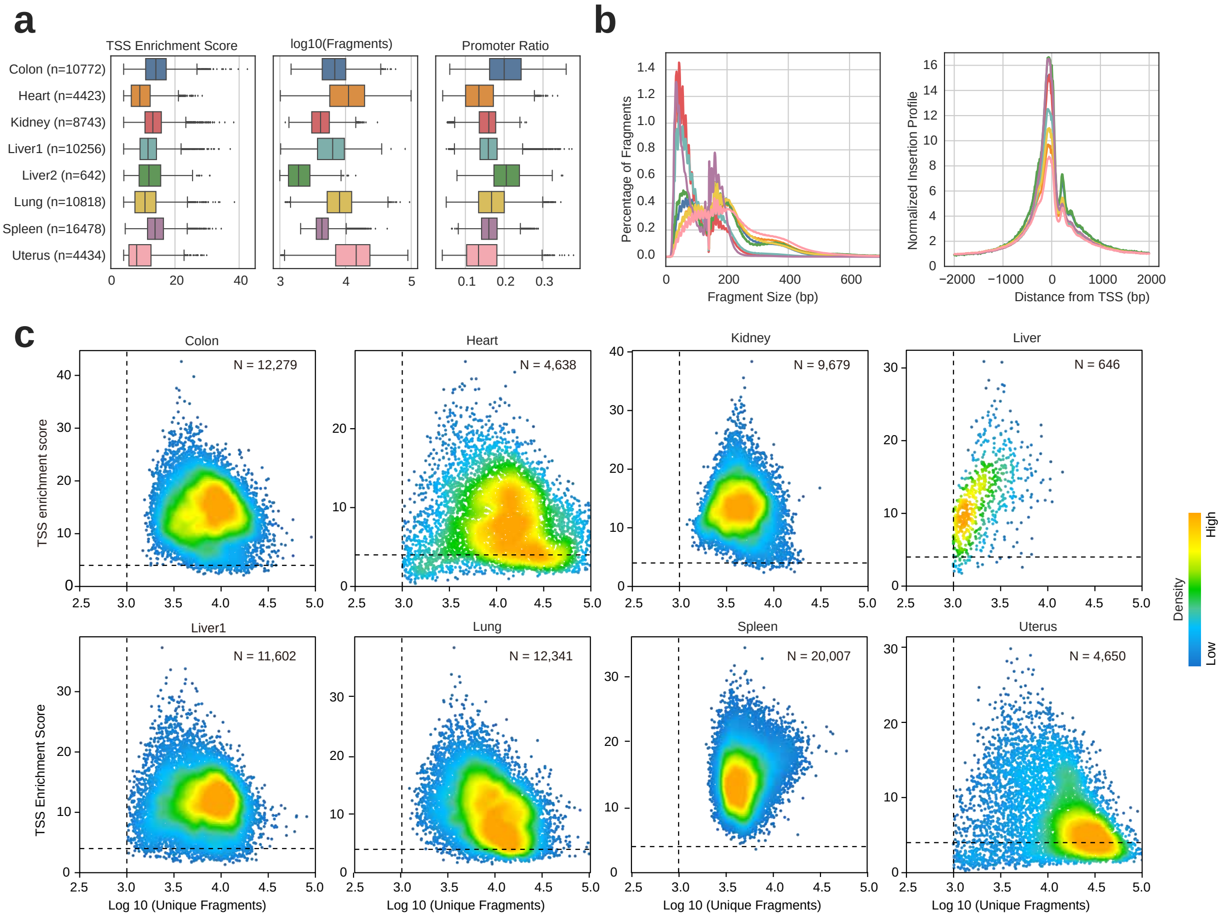

Supplementary Fig. 4. Quality control (QC) of scATAC-seq data. Related to Figure 1. **(a)** Box plot showing the distribution of TSS enrichment score, number of fragments and promoter ratio across eight samples. Each dot represents a cell. The boxes indicate the 25% quantile, median (horizontal line), 75% quantile and Tukey-style whiskers (beyond the box). **(b)** Fragment size distributions of eight samples (left). Aggregate TSS insertion profiles centred at all TSS regions. The showing cells are passing ArchR QC thresholds for each sample (right). The color code refers to **(a)**. **(c)** Scatter plot showing the TSS enrichment score vs unique nuclear fragments per cell. The colour of the dots represents the density of each point in the plot.

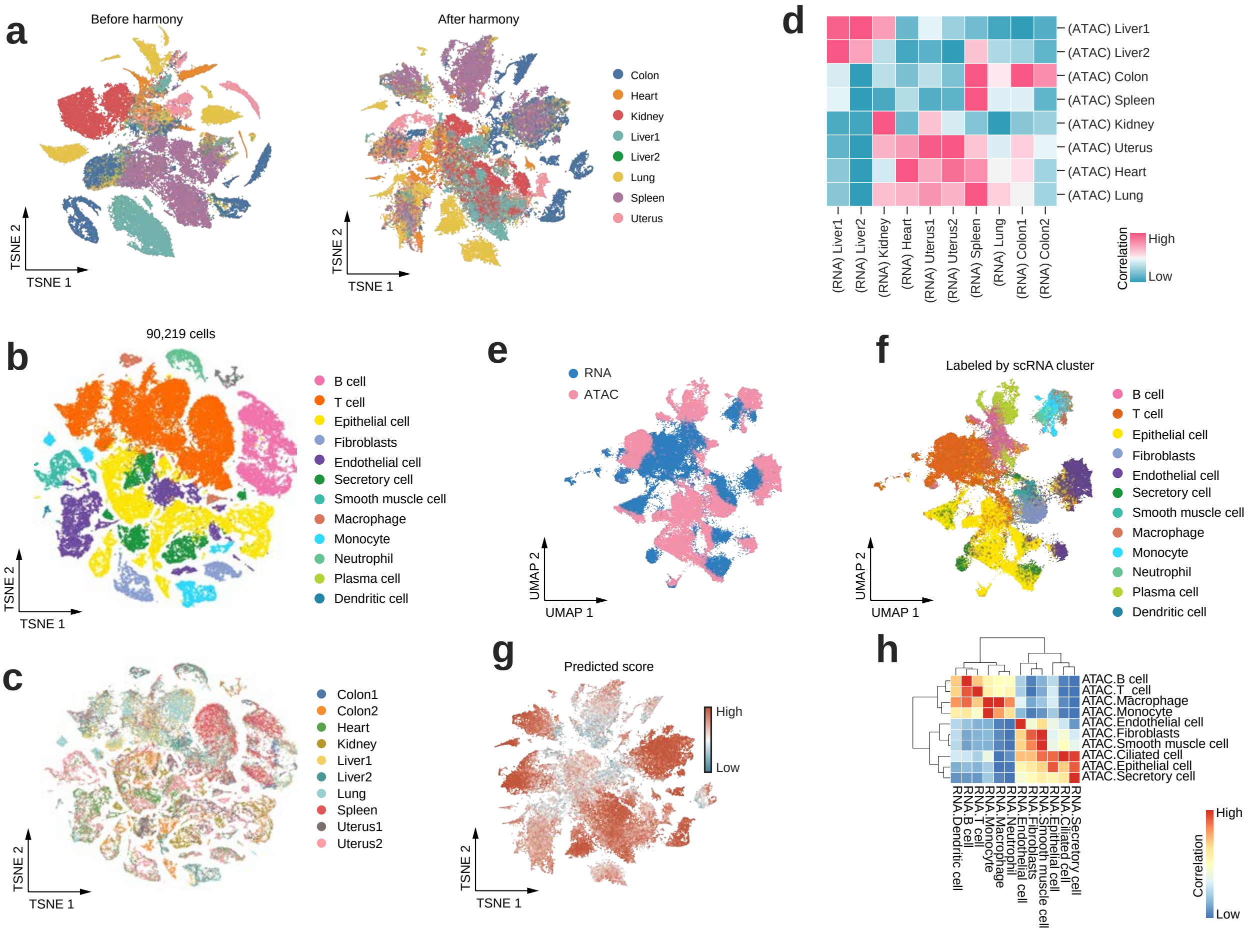

Supplementary Fig. 5. Cell type annotation based on scATAC-seq data. Related to Figure 1. **(a)** (left) t-SNE of ArchR iterative latent semantic indexing (LSI) and (right) ArchR iterative LSI with Harmony-based batch correction for all samples. **(b)** t-SNE embedding showing the subset of seven organs from scRNA-seq. Colored by cell type in **Fig.1b**. **(c)** t-SNE embedding showing the subset of seven organs from scRNA-seq data. Colored by organs. **(d)** Heatmap showing the spearman correlations between scATAC-seq and scRNA-seq data. Correlations are calculated from the top 5,000 expressed genes for each sample. **(e)** UMAP co-embedding of scATAC-seq and scRNA-seq cells. 40,000 cells with scATAC-seq and scRNA-seq were sampled according to the percentage of cell types. **(f)** Co-embedded cells colored by scRNA-seq cell-type annotations. **(g)** Scatter plot showing the predicted score distribution for scATAC-seq and scRNA-seq integration. **(h)** Heatmap showing the spearman correlations between scATAC-seq and scRNA-seq cell types. Correlations are calculated from the top 3,000 expressed genes for each cell type.

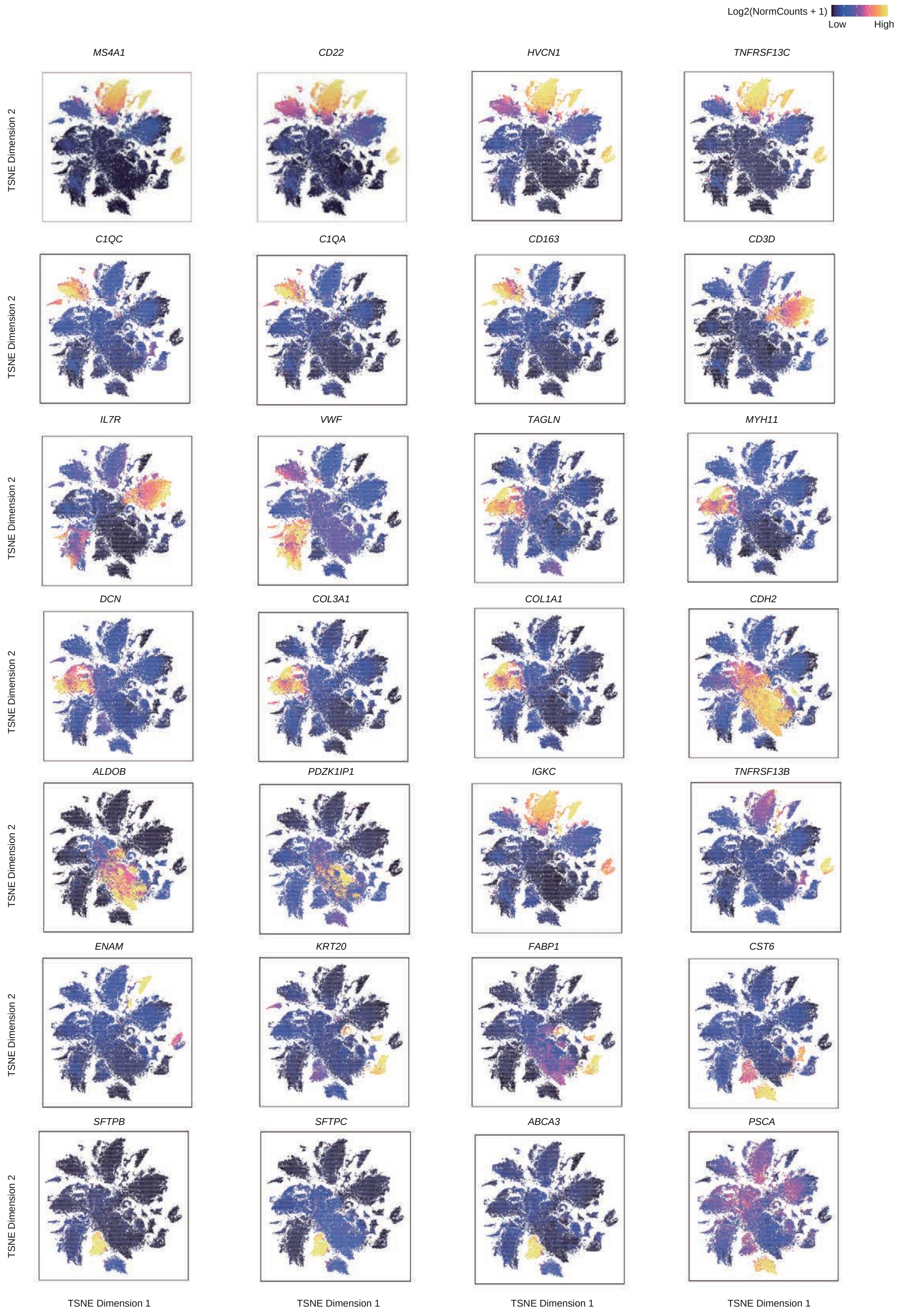

Supplementary Fig. 6. Marker genes expression based on scATAC-seq data. Related to Figure 1. Distribution of marker genes in cell types such as B cells (*MS4A1*, *CD22*, *HVCN1*, *TNFRSF13C*, *IGKC*, *TNFRSF13B*, *ENAM*), macrophages (*C1QC*, *C1QA*, *CD163*), T cells (*CD3D*, *IL7R*), endothelial cells (*VWF*), fibroblasts (*TAGLN*, *MYH11*, *DCN*, *COL3A1*, *COL1A1*), secretory cells (*CDH2*, *ALDOB*, *PDZK1IP1*), epithelial cells (*KRT20*, *FABP1*) and ciliated cells (*CST6*, *SFTPB*, *SFTPC*, *ABCA3*, *PSCA*) on the t-SNE plots.

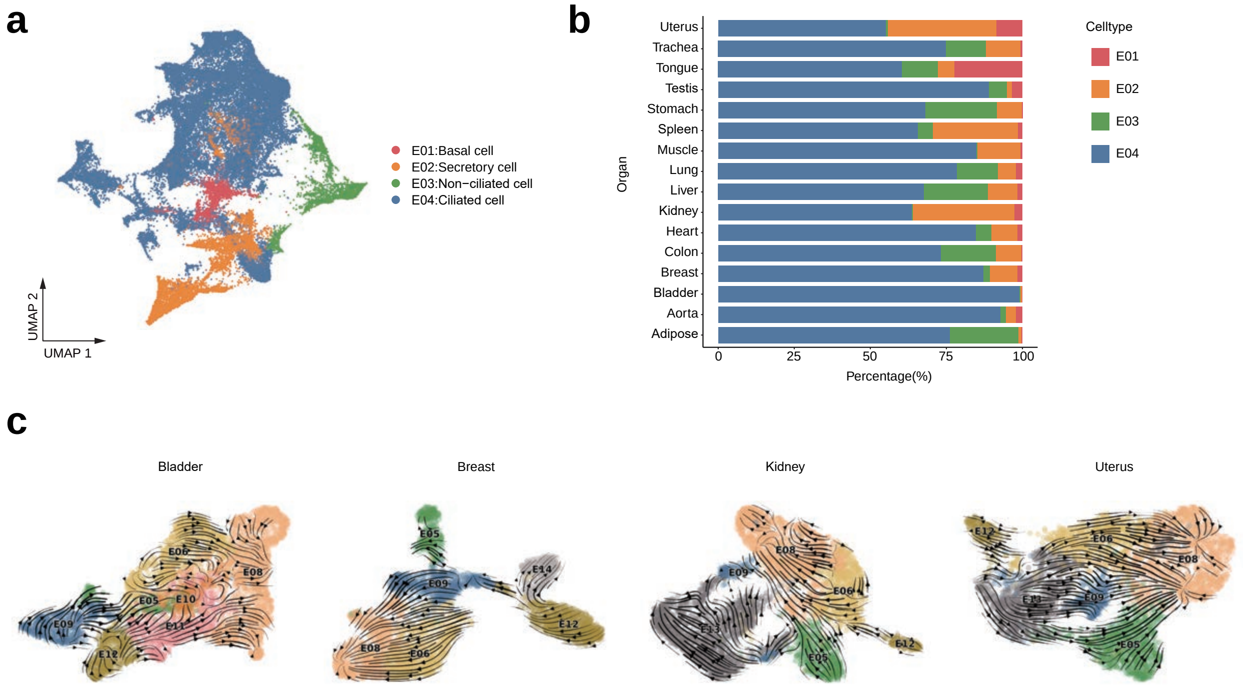

Supplementary Fig. 7. Pseudotime trajectory analysis by Monocle2. Related to Figure 2. **(a)** Distribution of the four epithelial cell subtypes on the UMAP. **(b)** Bar plot showing the percentage of cell subtypes in each organ. **(c)** RNA velocities of major ciliated cells in the organs of bladder, breast, kidney, and uterus. The tracks are colored by cell subtypes. The arrow indicates the direction of cell pseudotime differentiation.

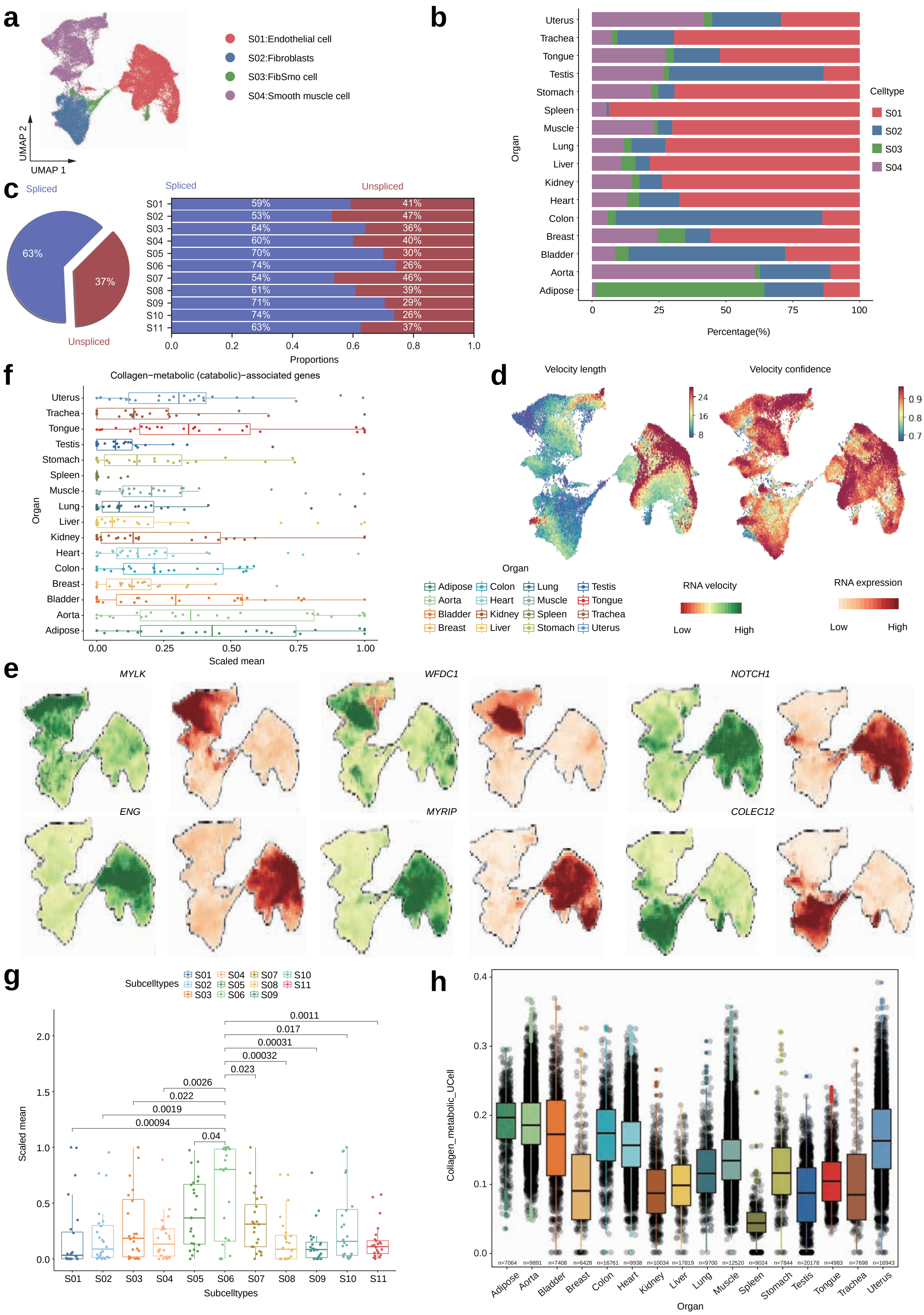
a
S01:Endothelial cell
S02:Fibroblasts
S03:FibSmo cell
S04:Smooth muscle cell
UMAP 1
UMAP 2
b
Celltype
S01
S02
S03
S04
Organ
Percentage(%)
c
Spliced
Unspliced
63%
37%
Proportions
d
Velocity length
Velocity confidence
RNA velocity
RNA expression
Low
High
e
MYLK
WFDC1
NOTCH1
ENG
MYRIP
COLEC12
f
Collagen−metabolic (catabolic)−associated genes
Scaled mean
g
Subcelltypes
Scaled mean
h
Collagen_metabolic_UCell
Organ

Supplementary Fig. 8. Pseudotime trajectory analysis by RNA velocity. Related to Figure 3. **(a)** Distribution of the four stromal cell subtypes on the UMAP. **(b)** Bar plot showing the percentage of cell subtypes in each organ. **(c)** Pie chart showing the proportion of stromal cells that are spliced versus un-spliced. Bar plot showing the proportion of stromal cell subtypes that are spliced versus un-spliced. **(d)** UMAP diagrams showing the velocity length and velocity confidence of stromal cells by RNA velocity analysis. **(e)** Distribution of marker genes on the UMAPs based on RNA velocity analysis. **(f-g)** Box plot showing the mean expression of collagen metabolic (catabolic) associated genes ($n = 24$) in cells from different organs and subcelltypes (Mann-Whitney test, $p\text{-value} < 0.05$). Each point indicates a gene. The boxes indicate the 25% quantile, median (horizontal line), 75% quantile. **(h)** Box plot showing the distribution of gene signature scores estimated by UCell based on annotated genes ($n = 24$) from the collagen metabolic pathway. The boxes indicate the 25% quantile, median (horizontal line), 75% quantile and Tukey-style whiskers (beyond the box).

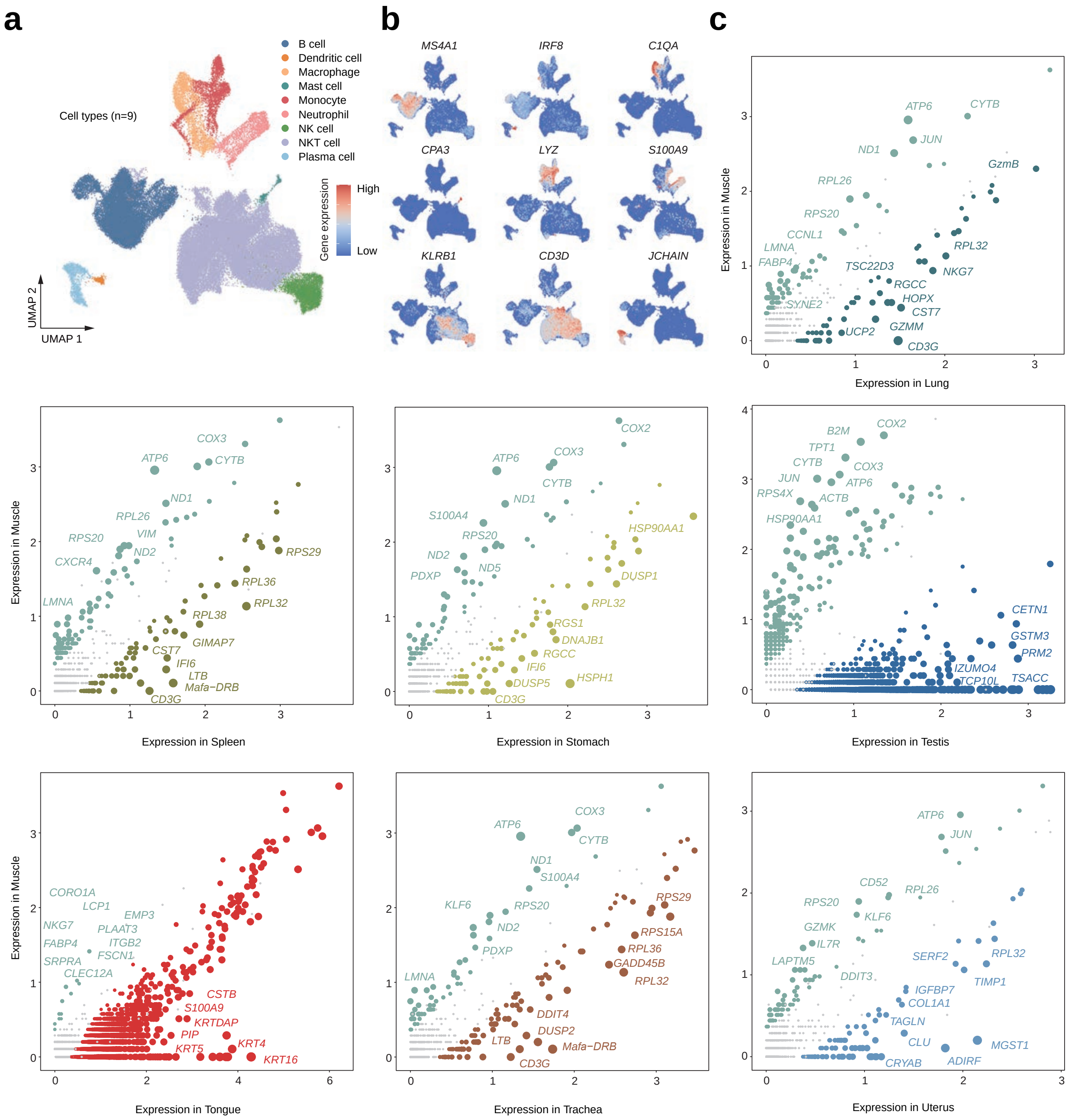

Supplementary Fig. 9. Differentially expressed gene (DEG) analysis of the immune cell subsets. Related to Figure 4. **(a)** Distribution of the nine immune cell subtypes on the UMAP. **(b)** Feature plot showing the expression of marker genes. **(c)** Scatter plot showing the comparison of gene expression between muscle and other organs. The size of dots is proportionate to the fold change of genes.

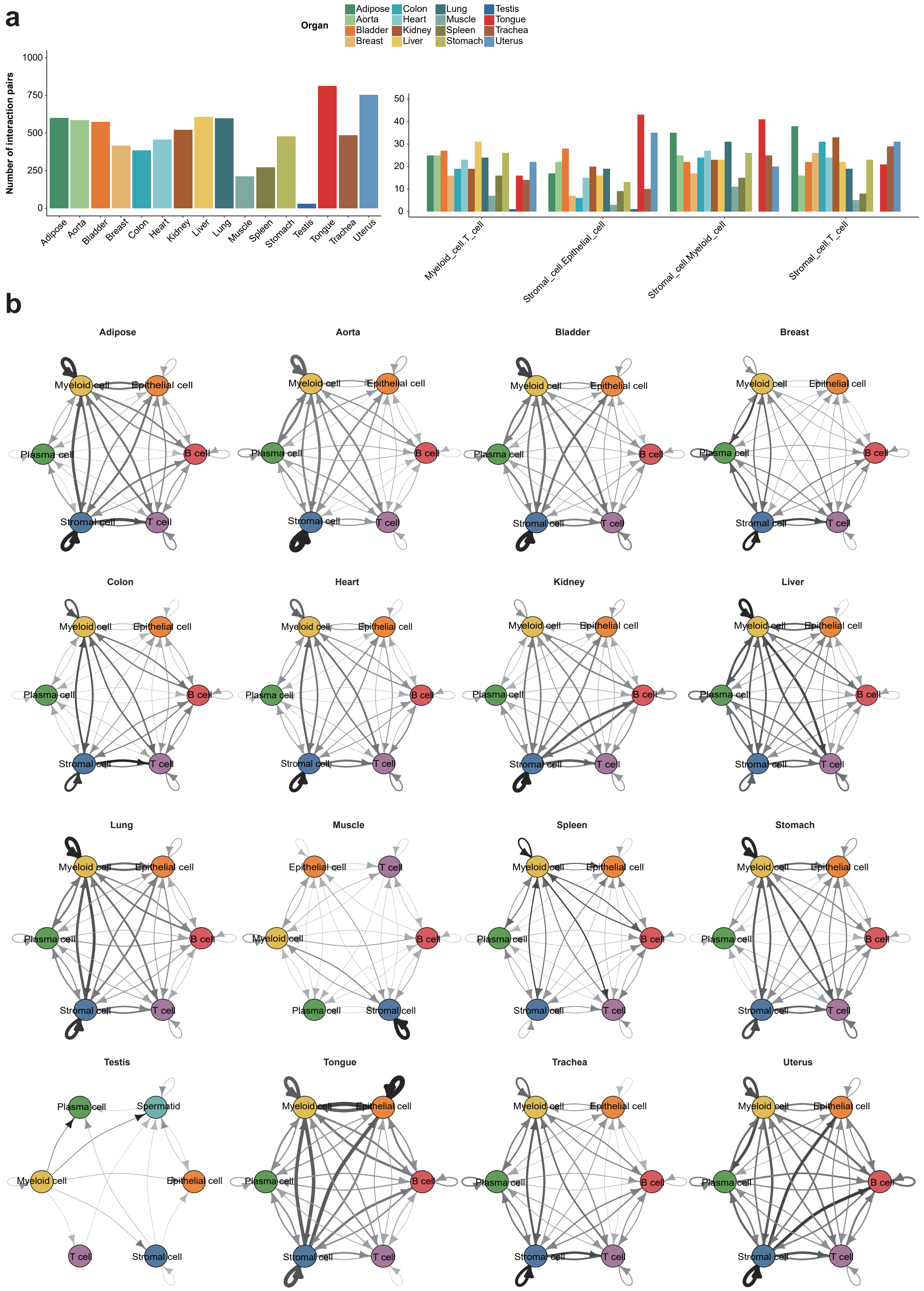

Supplementary Fig. 10. Cell-cell interaction analysis. Related to Figure 5. **(a)** Bar chart showing the number of interaction pairs in organs (left) or by cell interaction types (see **Fig. 5d**) (right). **(b)** Network diagrams showing cell-cell interactions among major cell types in the 16 organs. Line thickness represents the strength of cell-cell interactions.

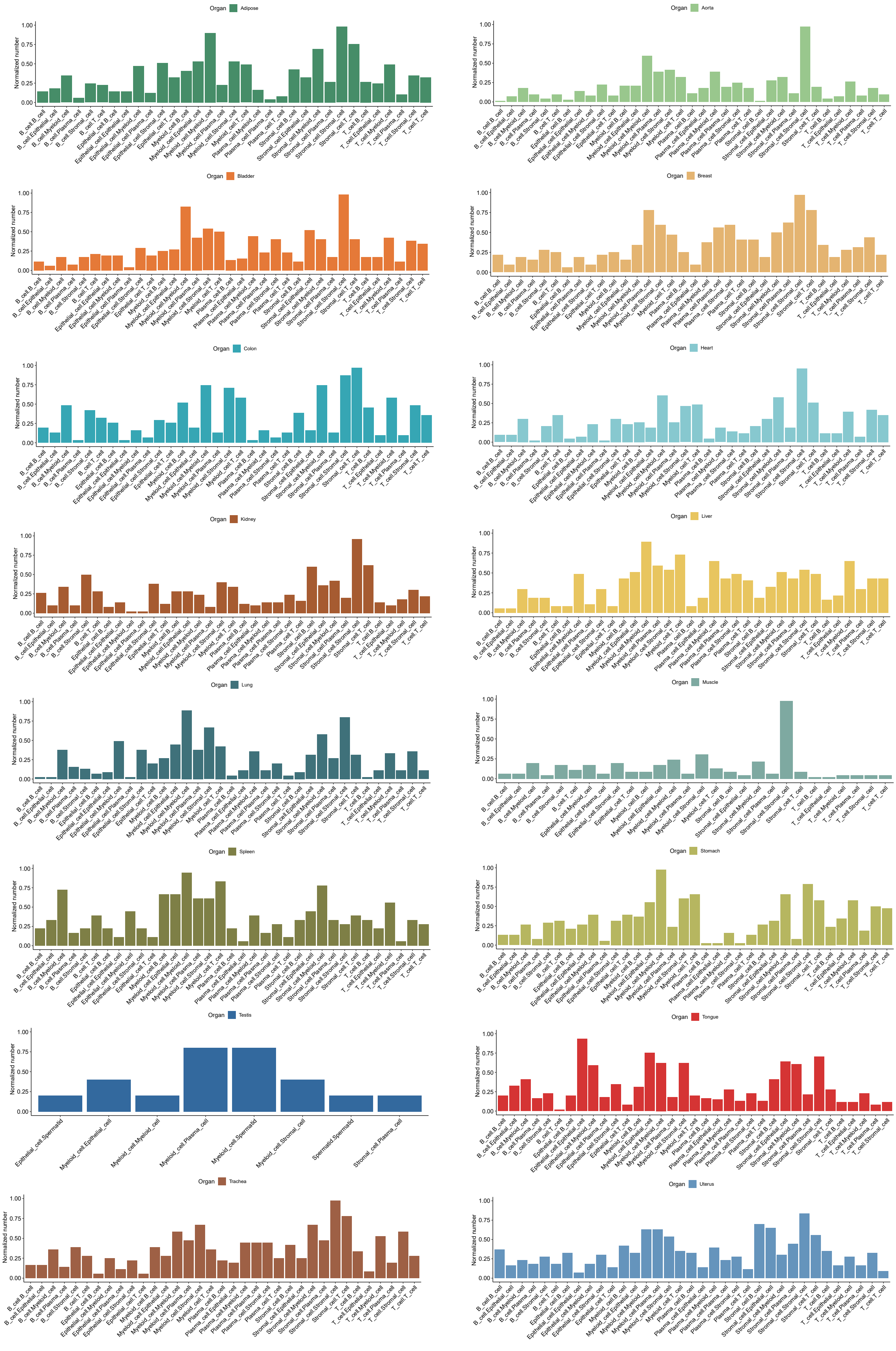

Supplementary Fig. 11. Statistical analysis of cell-cell interactions. Related to Figure 5. Bar chart showing the normalized number of interaction pairs of different cell-cell interaction types in the 16 organs.

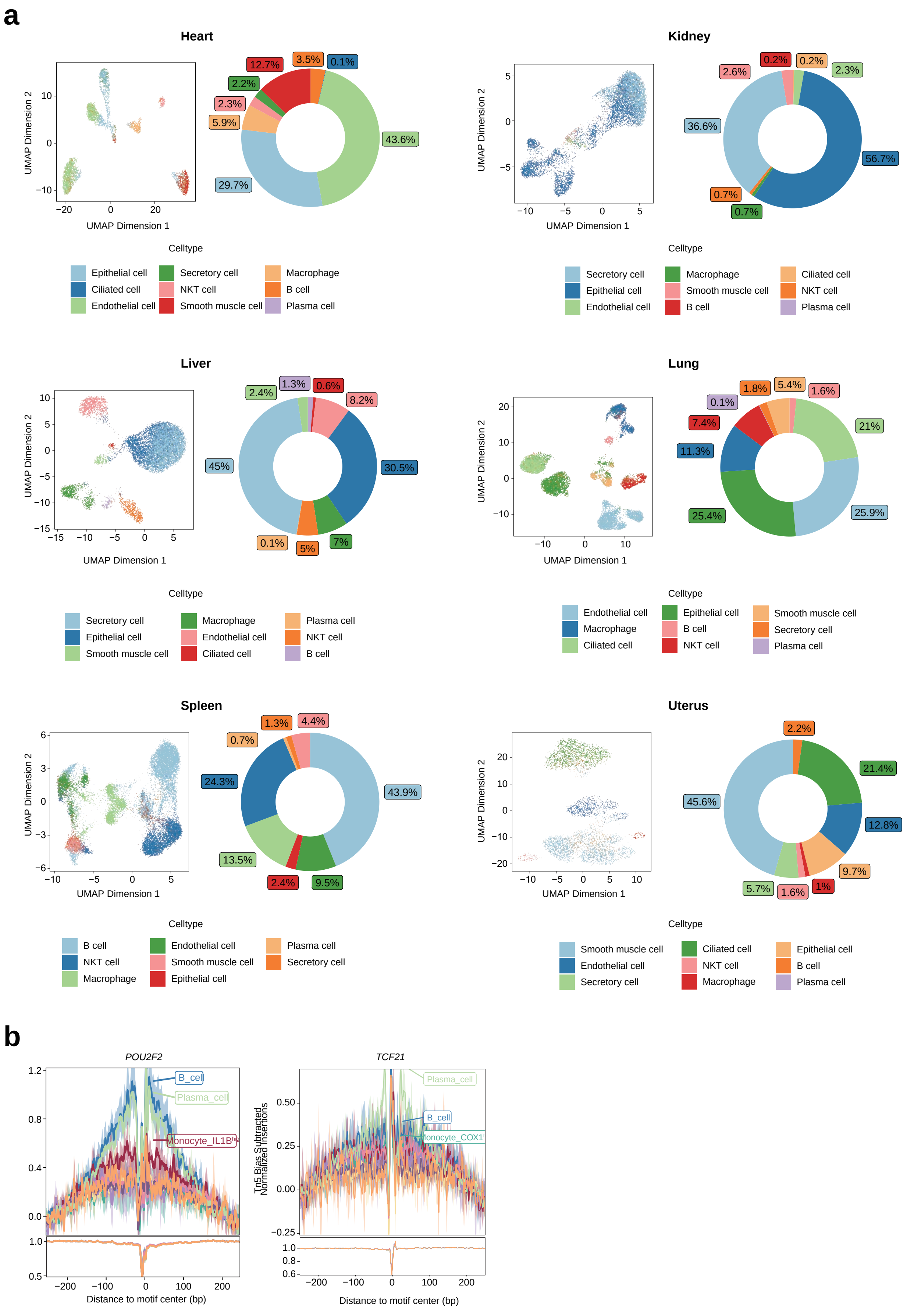

Supplementary Fig. 12. Cell type annotation based on scATAC-seq in each organ. Related to Figure 6. (a) Distribution of cells on the UMAP in the six organs, and donut chart showing the proportion of cells over cell types. **(b)** Tn5 bias-adjusted TF footprint analysis of the *POU2F2* and *TCF21* transcription factors.

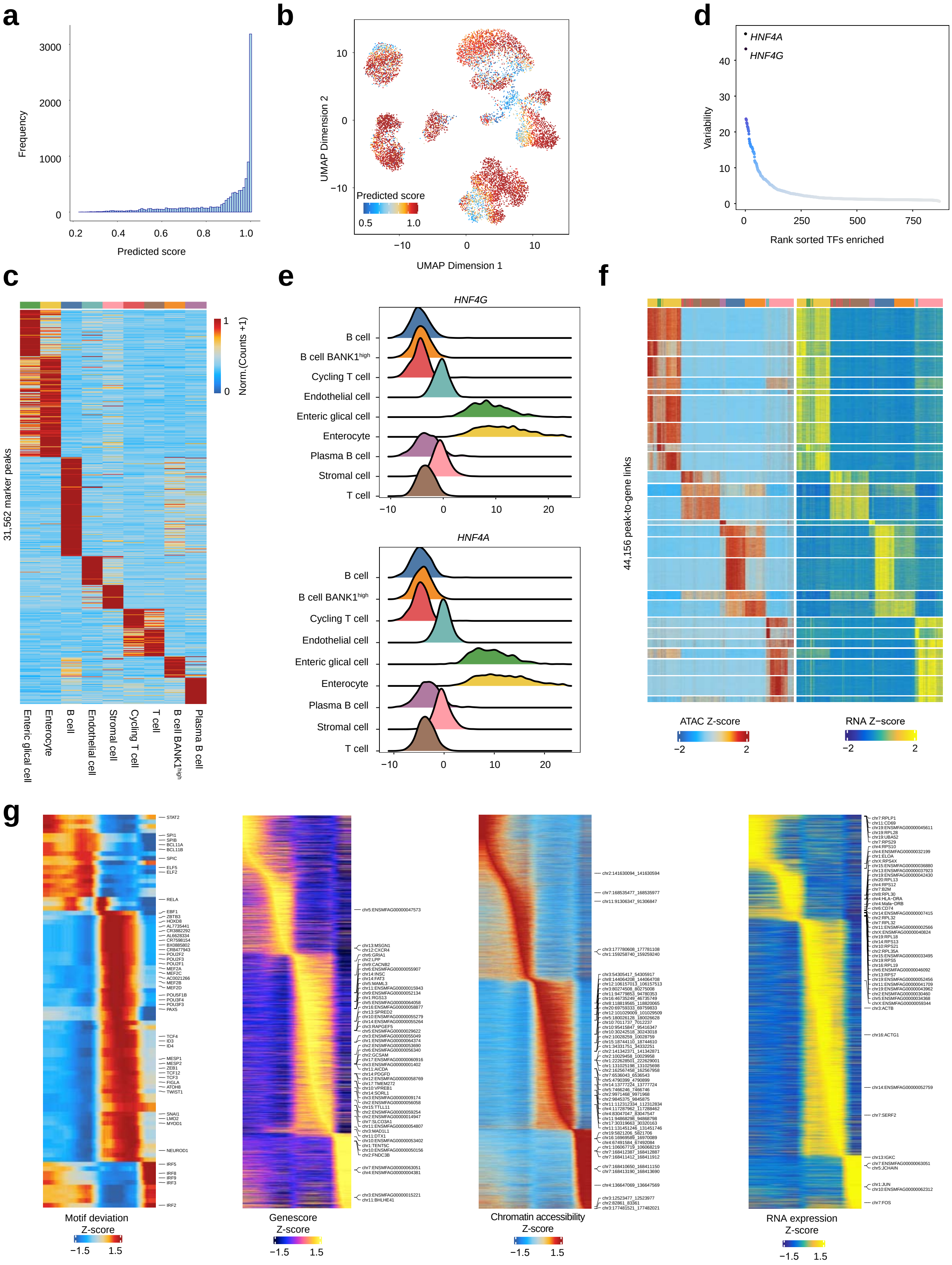

Supplementary Fig. 13. scATAC-seq data analysis in colon. Related to Figure 7. **(a)** Distribution of cell type prediction score for scATAC-seq data based on matched scRNA-seq data. The score ranges from 0 to 1, and a higher score indicates a higher prediction confidence. **(b)** Distribution of prediction score in UMAP embeddings. **(c)** Heatmap showing row-normalized chromatin accessibility of 31,562 cell type-specific peaks (FDR < 0.1, log2FC > 3). **(d)** The scatter plot showing the ranked differential motif enrichment in colon cell types. **(e)** The ridgeline plot showing the chromVAR TF deviation scores of *HNF4G* and *HNF4A* in each cell types. **(f)** The heatmap showing chromatin accessibility and gene expression of 44,156 significantly ($R > 0.45$ and FDR < 0.01) linked peak-gene pairs. **(g)** The heatmap showing the dynamic of motif deviation score, gene-score, chromatin accessibility, and RNA expression across the B cell psedotime trajectory.

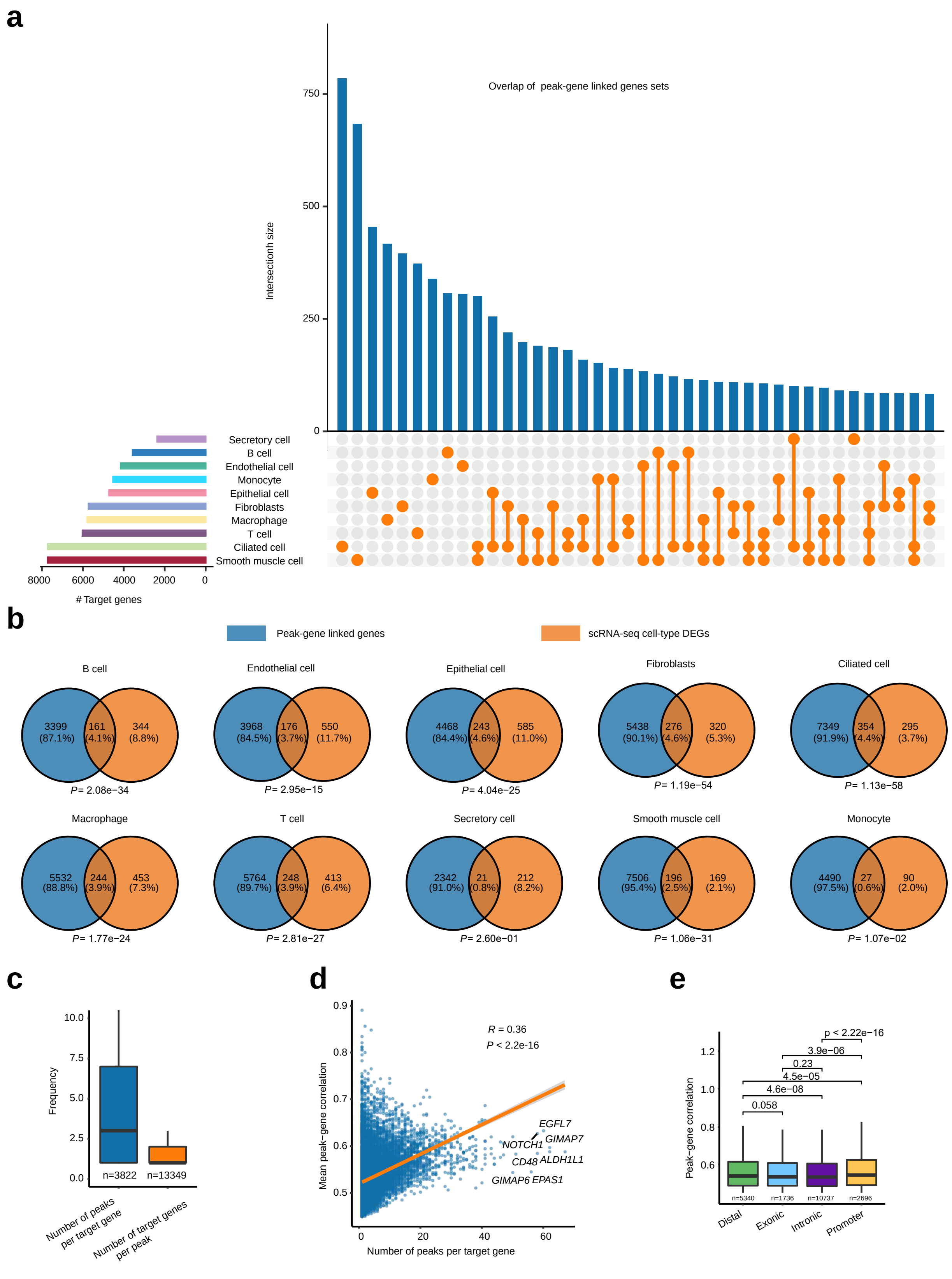

Supplementary Fig. 14. Statistics of peak-gene links. Related to Figure 7. **(a)** Upset plot showing the size of overlaps between the sets of peak-gene linkes identified in each cell type. The bar plot on the left shows the set size of peak-gene links for each cell type, and the bar plot on the top shows the number of overlapping genes between two sets, or the number of unique genes in one set. **(b)** Venn diagrams for each cell type showing the overlaps between the set of peak-gene linked genes and differential expressed genes (inferred by scRNA-seq, log2FC $>$ 1, FDR $<$ 0.01) in this cell type. A hypergeometric test was used for gene-set overlap significance. **(c)** The boxplot showing the number of peaks per target gene and target genes per peak. The boxes indicate the 25% quantile, median (horizontal line), 75% quantile. **(d)** The scatter showing the correlation between the number of peaks per target gene and the peak-gene links correlation. Some genes with more peaks are labed. P-values are provided (two-sided Spearman's correlation test). Fitted line and standard errors with 95% confidence intervals are shown. **(e)** The boxplot showing the peak-gene correlation (two-sided Mann–Whitney U test) distribution of distal, exonic, intronic, and promoter. The boxes indicate the 25% quantile, median (horizontal line), 75% quantile.

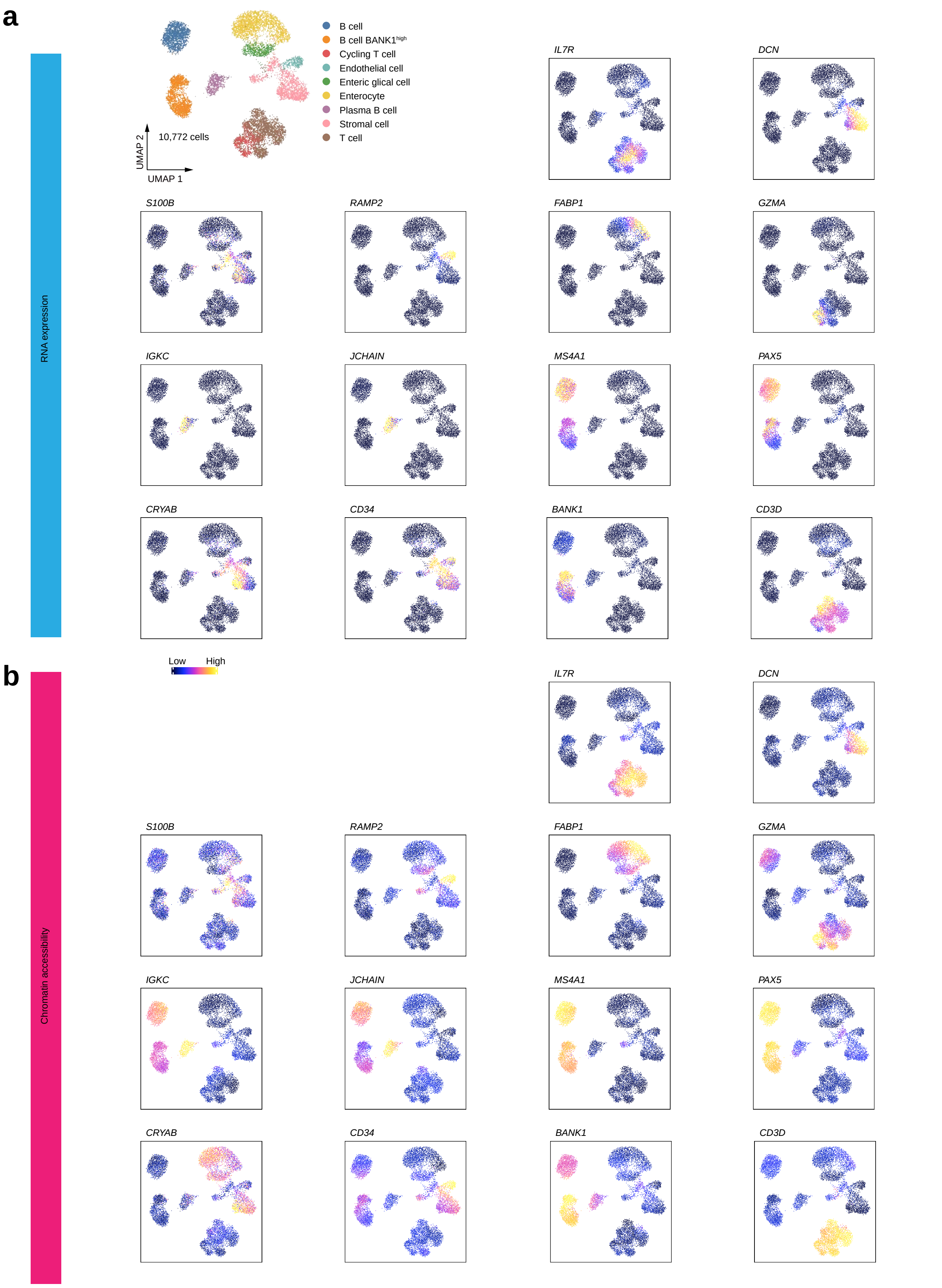

Supplementary Fig. 15. A supplement for scATAC-seq data analysis in colon. Related to Figure 7. UMAP plots for the colon cells colored by **(a)** gene expression from scRNA-seq alignment or **(b)** chromatin accessibility for key marker genes.

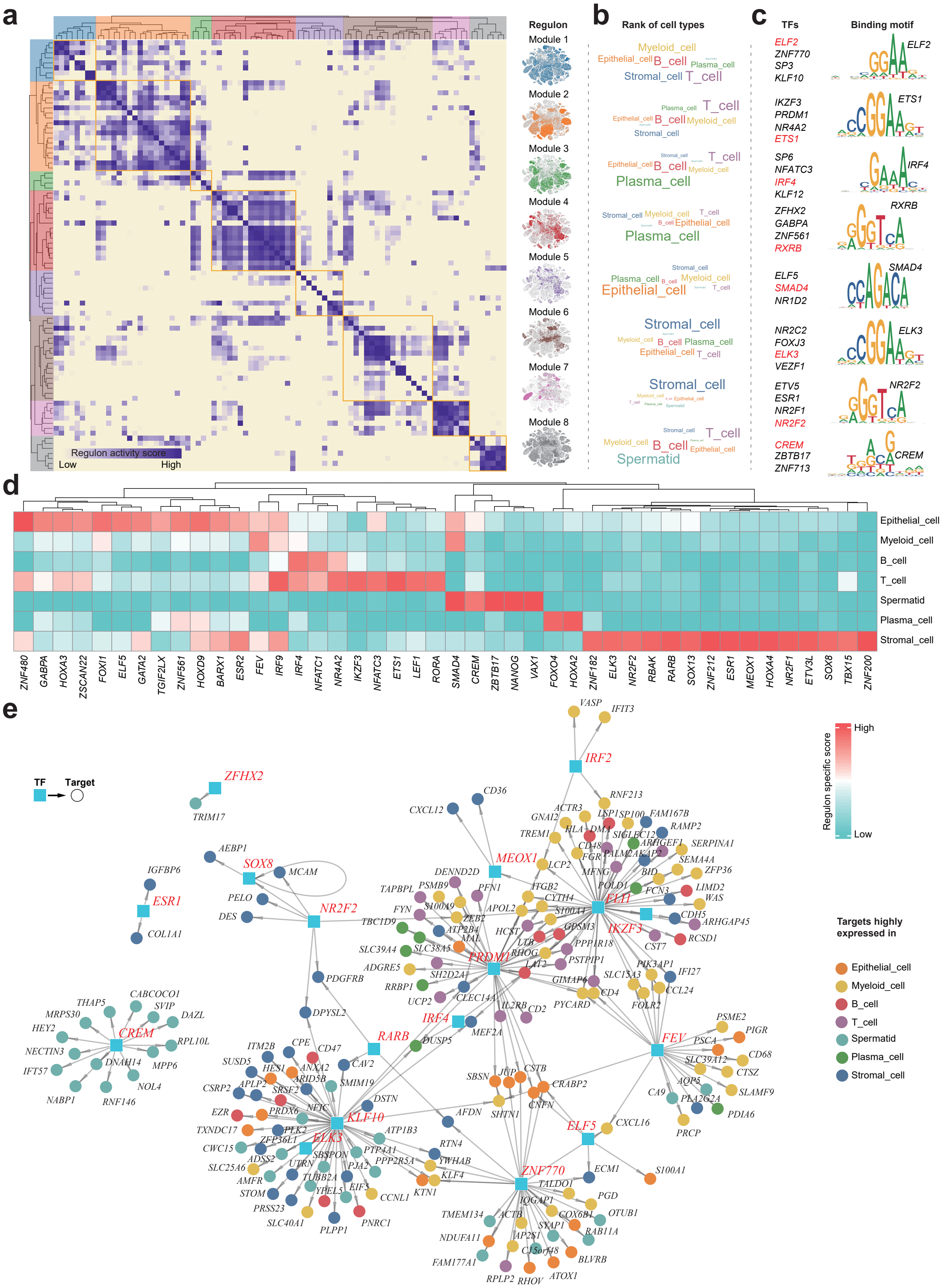

Supplementary Fig. 16. Gene regulatory network analysis in cynomolgus monkey. Related to Figure 8. **(a)** Identification of regulon modules using SCENIC. Heatmap (left) shows the similarity of different regulons (n=86) based on the AUCell score. Eight regulon modules were identified based on regulon similarity. UMAPs (right) illustrate the average AUCell score distribution for different regulon modules (in different colors). **(b)** Wordcloud plots showing the enrichment of cell types in different regulon modules. **(c)** Representative transcription factors and corresponding binding motifs in different regulon modules. **(d)** Heatmap showing transcription factors enriched in different cell types. Color depth represents the level of regulon-specific score. **(e)** Integrated gene-regulatory networks of the regulons from **(a)**. Regulon-associated TFs are highlighted in blue rectangles and target genes in circles. Target genes (in circles) are colored according to their highly expressed cell types.

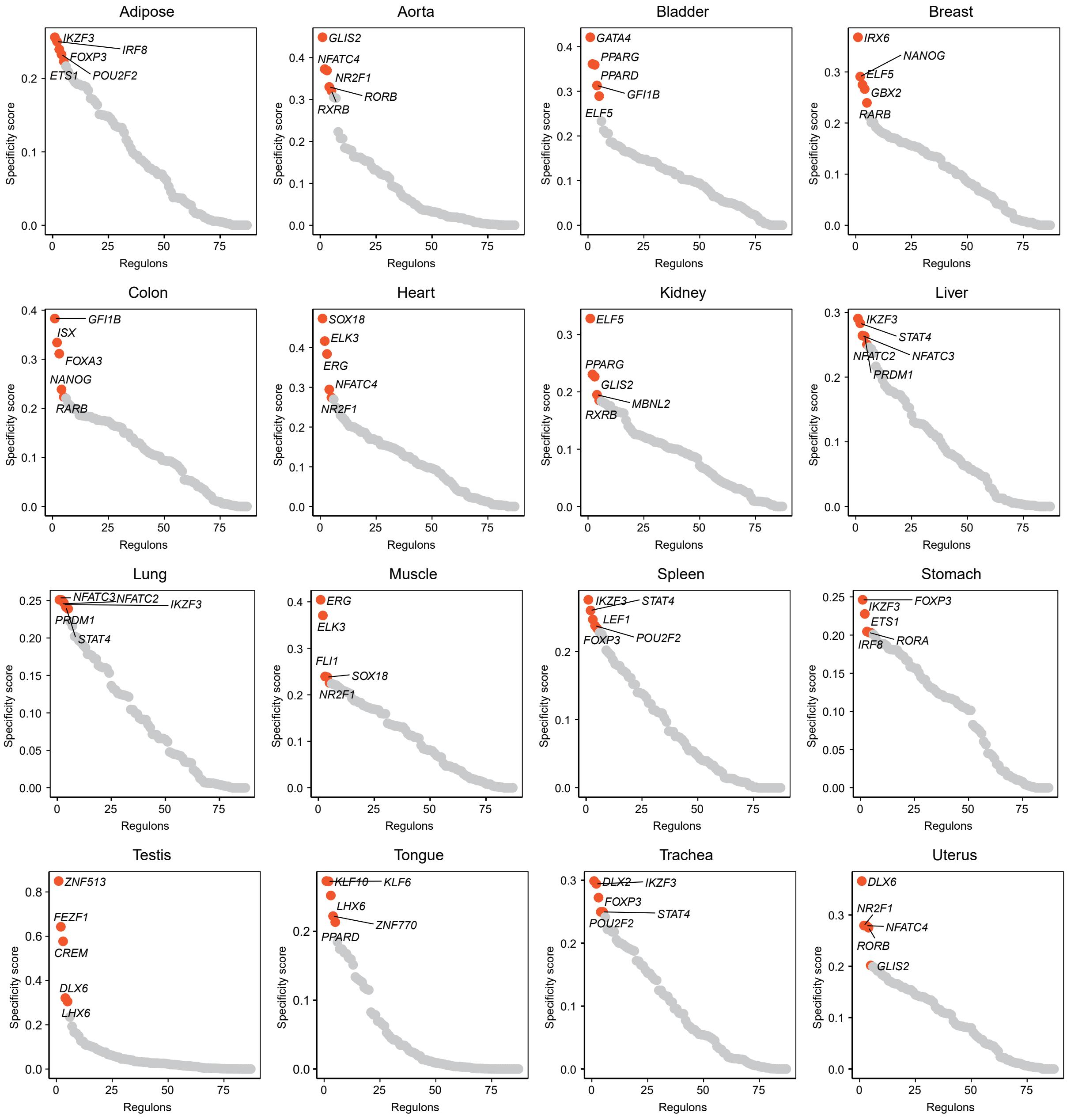

Supplementary Fig. 17. Regulons and modules for organs. Related to Figure 8. Scatter plot showing top regulons of 16 organs ordered by regulon specific score and top regulons with high activity are highlighted.

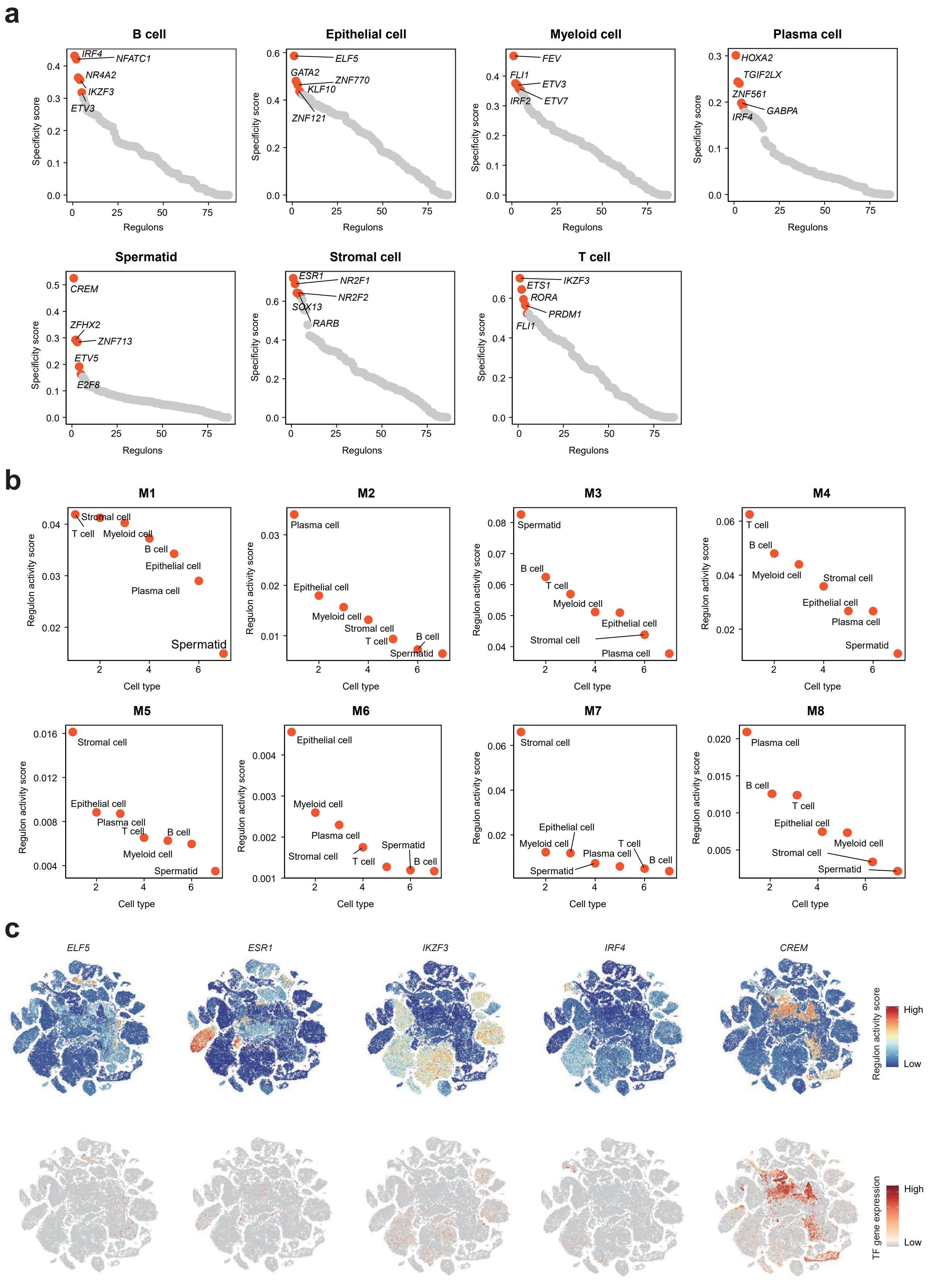

Supplementary Fig. 18. Regulons and modules for cell types. Related to Figure 8. **(a)** Scatter plot showing top regulons of the seven major cell types ordered by regulon specific score and top regulons with high activity are highlighted in red. **(b)** Scatter plot showing major cell types of the eight regulon modules ordered by regulon activity score. **(c)** t-SNE plots displaying the AUCell score distribution for the selected regulons (top) and gene expression patterns for the corresponding TFs (bottom).

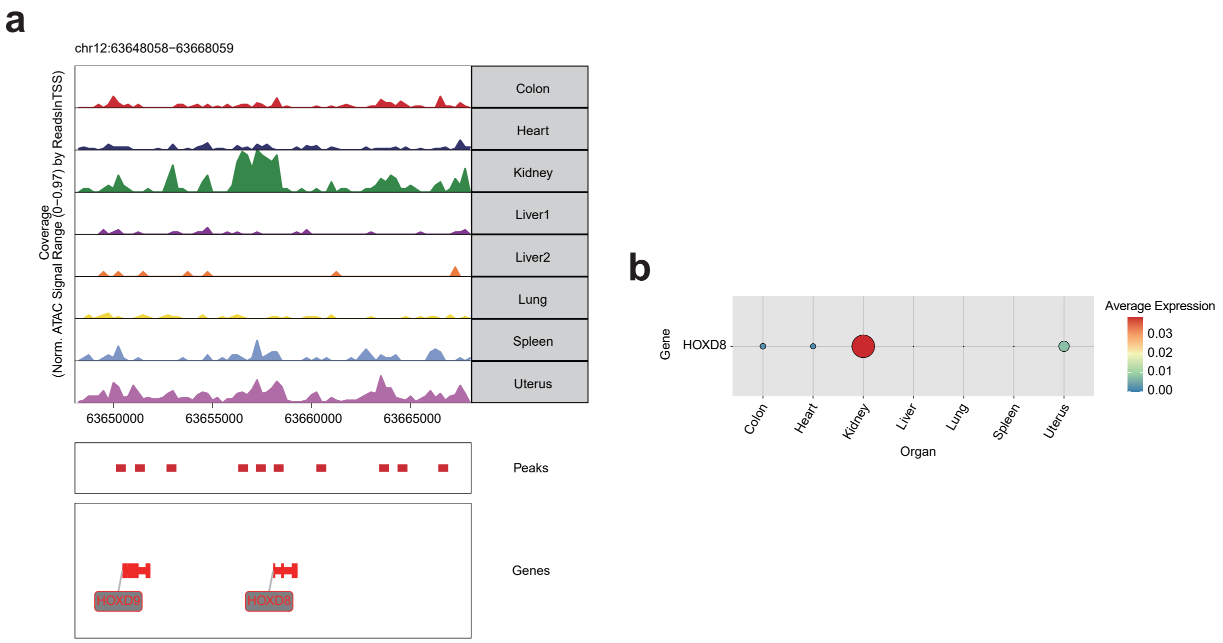

Supplementary Fig. 19. The CRE accessibility and the expression level of the gene *HOXD8*. Related to Figure 8. **(a)** Genome browser tracks of the local chromatin accessibility at the locus of *HOXD8* on a per tissue basis using ArchRBrowser (chr12:63,648,05863,668,059). **(b)** Dot plot shows the expression of *HOXD8* in different organs.

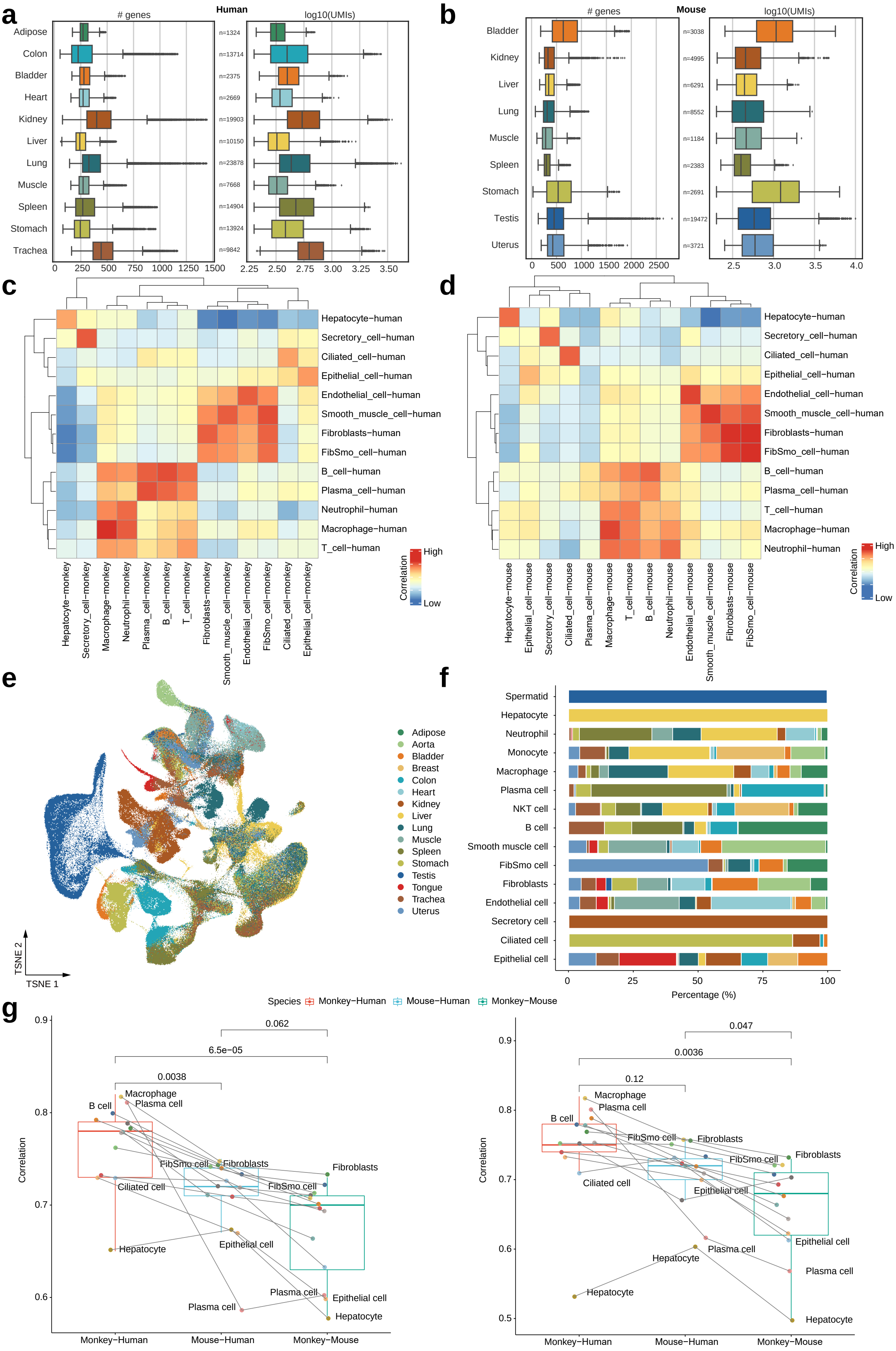
a
b
c
d
e
f
g
Human
Mouse
# genes
log10(UMIs)
Adipose
Colon
Bladder
Heart
Kidney
Liver
Lung
Muscle
Spleen
Stomach
Trachea
Testis
Uterus
Hepatocyte−human
Secretory_cell−human
Ciliated_cell−human
Epithelial_cell−human
Endothelial_cell−human
Smooth_muscle_cell−human
Fibroblasts−human
FibSmo_cell−human
B_cell−human
Plasma_cell−human
Neutrophil−human
Macrophage−human
T_cell−human
Correlation
High
Low
TSNE 1
TSNE 2
Aorta
Breast
Tongue
Percentage (%)
Species
Monkey−Human
Mouse−Human
Monkey−Mouse

Supplementary Fig. 20. Data quality control for across-species analysis. Related to Figure 9. **(a)** Box plot showing the number of genes and UMIs in human organs. The boxes indicate the 25% quantile, median (horizontal line), 75% quantile and Tukey-style whiskers (beyond the box). **(b)** Box plot showing the number of genes and UMIs in mouse organs. The boxes indicate the 25% quantile, median (horizontal line), 75% quantile and Tukey-style whiskers (beyond the box). **(c)** Heatmap showing the Spearman correlation of top gene expression in cell types between cynomolgus monkey and human. **(d)** Heatmap showing Spearman correlation of top genes expression in cell types between mouse and human. **(e)** Distribution of cynomolgus monkey, human and mouse cells by organs on the UMAP. **(f)** Bar plot showing the percentage of cell types in each organ. **(g)** Box plots showing the Spearman correlation of average gene expression between two different species using top marker genes (top 100 and top 1500) in a specific cell type (t.test). Each dot represents a major cell type. The boxes indicate the 25% quantile, median (horizontal line), 75% quantile.

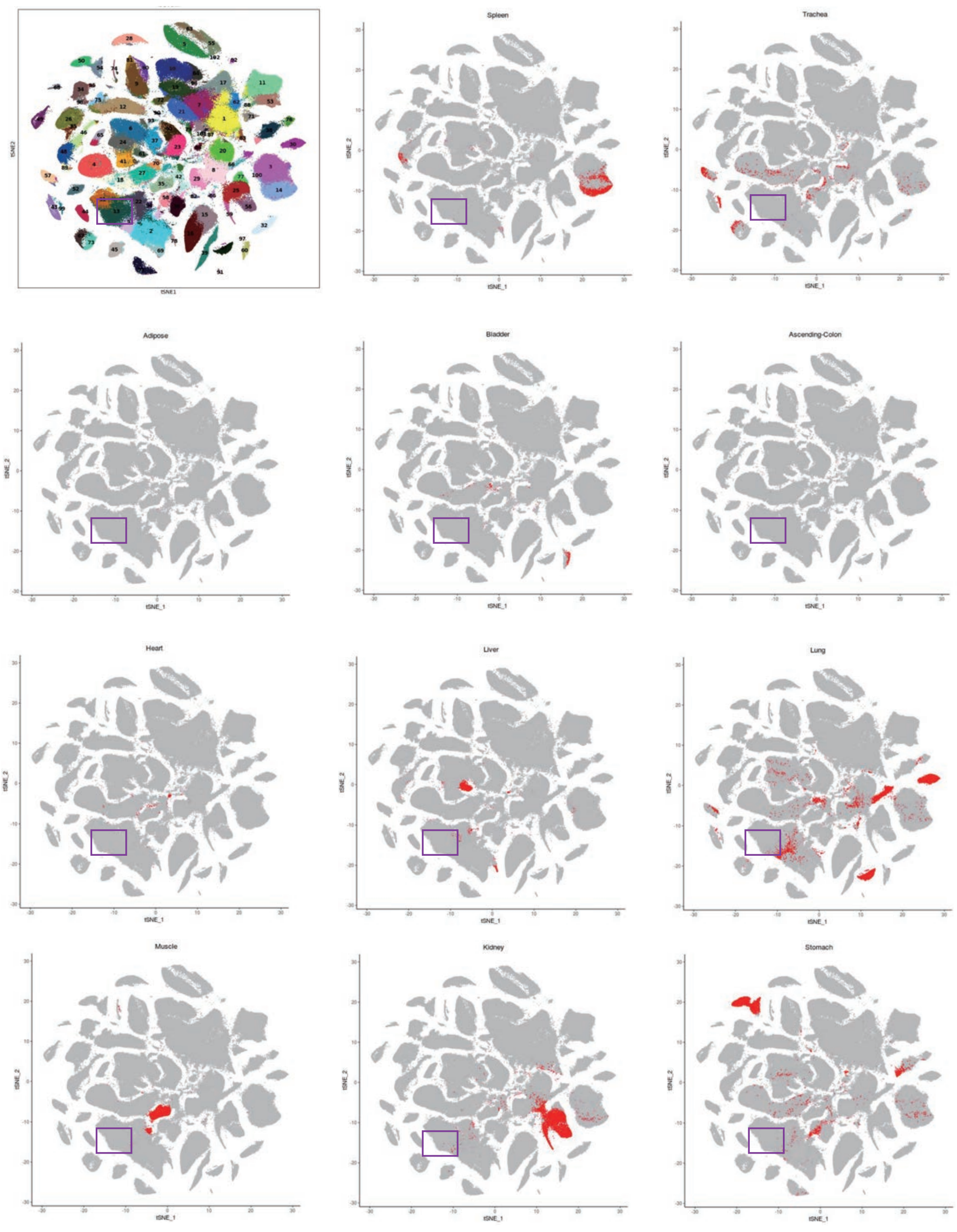

Supplementary Fig. 21. Validation of cell type annotation in across-species analysis. Related to Figure 9. The first t-SNE diagram shows the annotation of cell clusters in human (Han et al. Nature 2020). The cluster 13 is annotated as monocyte. Other t-SNE plots indicates cells (in red) from a specific organ. The purple boxes indicate where monocytes are located.

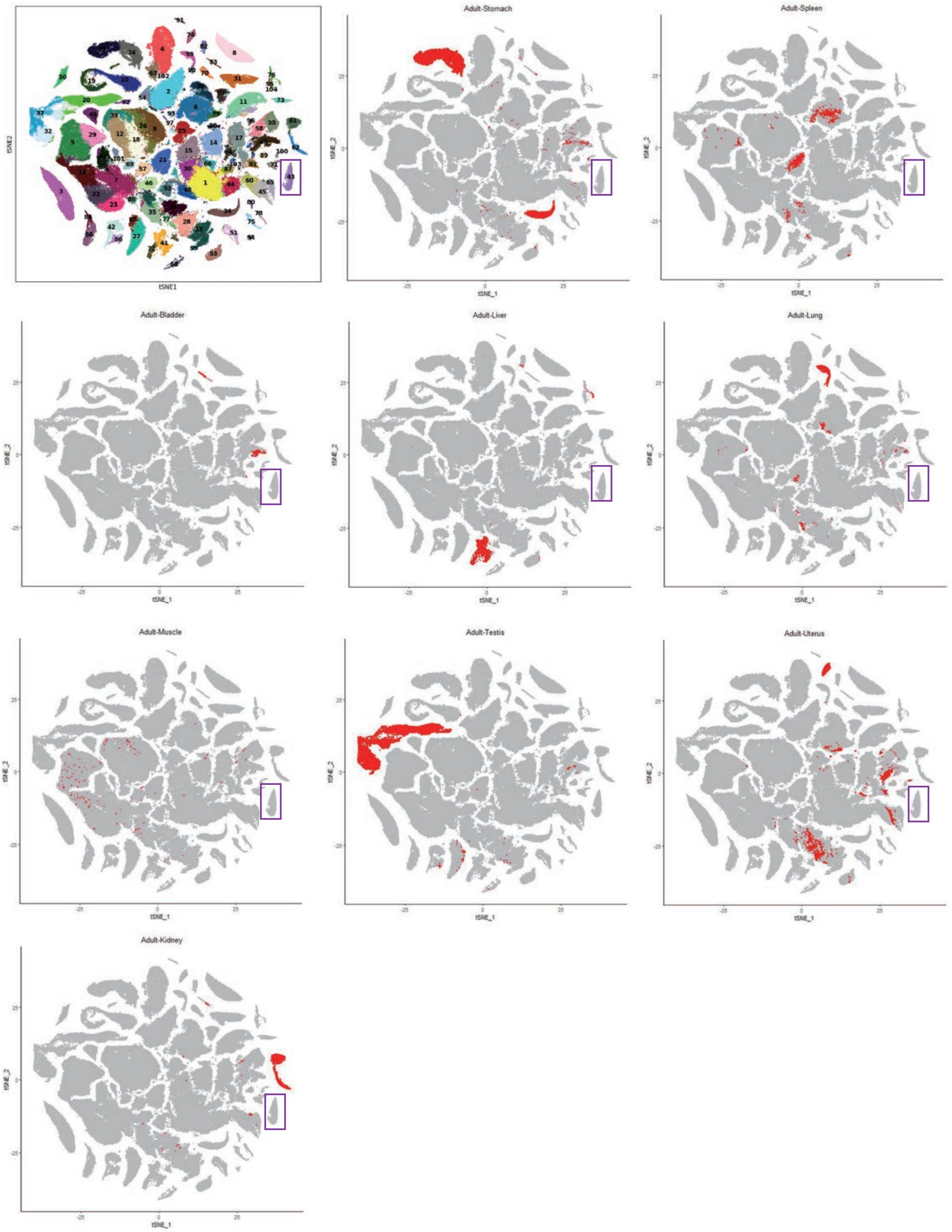

Supplementary Fig. 22. Validation of cell type annotation in across-species analysis. Related to Figure 9. The first t-SNE diagram shows the annotation of cell clusters in mouse (Han et al. Cell 2018). The cluster 43 is annotated as monocyte. Other t-SNE plots indicates cells (in red) from a specific organ. The purple boxes indicate where monocytes are located.

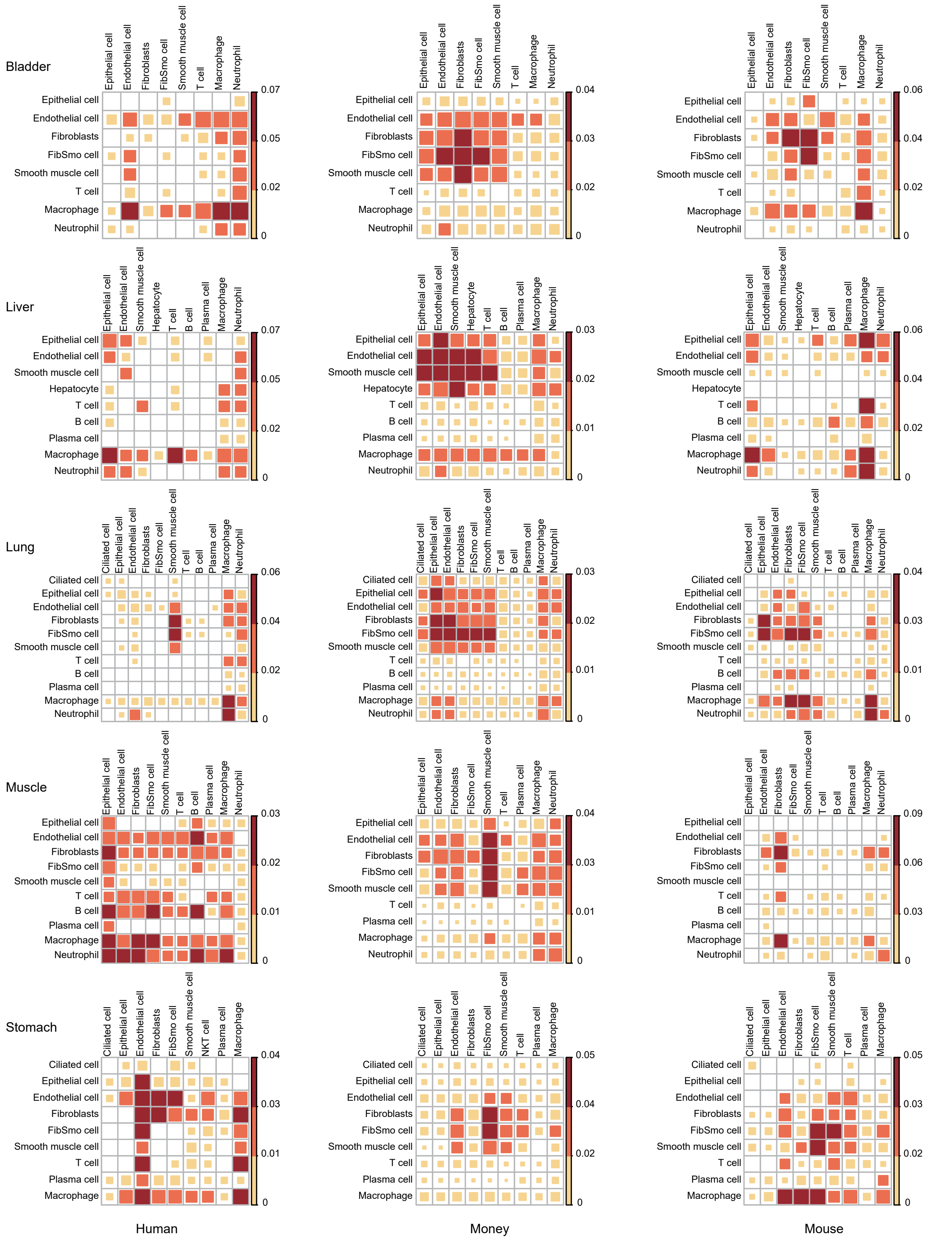

Supplementary Fig. 23. Across-species comparisons of cell-cell interaction analyses. Related to Figure 10. The heatmaps showing the strength of interactions among the common major cell types in bladder, liver, lung, muscle and stomach in human, mouse and monkey. The size and color of the blocks are proportional to the frequency of interactions. P-values were calculated by CellPhoneDB without multiple comparisons.

Supplementary Tables

Supplementary Table 1. The information of cynomolgus monkeys

Sex	Age	Health	Experimental history	Diet	Environment	Organ
Female	5 years old	Health	No	The animals were given water ad libitum and fed daily with 70 g commercially available solid food and 100 g apples.	The environment of the animal room was set at 25 ± 2°C room temperature, 60 ±5% relative humidity, and a 12 h light-and-dark cycle.	scRNA-seq (n=6): Uterus-1, Uterus-2,Lung, Breast, Liver, Colon<br><br>scATAC-seq(n=5): Uterus, Lung, Liver, Heart, Colon
Male	5 years old	Health	No	The animals were given water ad libitum and fed daily with 70 g commercially available solid food and 100 g apples.	The environment of the animal room was set at 25 ± 2°C room temperature, 60 ±5% relative humidity, and a 12 h light-and-dark cycle.	scRNA-seq(n=14): Trachea, Spleen, Stomach; Kidney, Tongue, Testis-1, Testis-2, Muscle, Bladder, Adipose, Aorta, Liver, Heart, Colon<br><br>scATAC-seq(n=3): Spleen; Kidney, Liver

Supplementary Table 2. Annotation information for cell types

Cluster	Minor cell type	Major cell type	Marker
C0	B cell	B cell	*MS4A1*
C1	NKT cell	T cell	*CD3D*
C2	Epithelial cell	Epithelial cell	*UBE2C*
C3	NKT cell	T cell	*CD3D*, *IL7R*
C4	Fibroblasts	Stromal cell	*PI16*, *APOD*, *GSN*
C5	Spermatid	Spermatid	*TMEM190*
C6	Endothelial cell	Stromal cell	*VWF*
C7	Epithelial cell	Epithelial cell	*KRT19*, *FABP1*, *KRT20*
C8	NKT cell	T cell	*CD3D*, *IL7R*
C9	NKT cell	T cell	*CD3D*
C10	Endothelial cell	Stromal cell	*VWF*
C11	Secretory cell	Epithelial cell	*PDZK1IP1*, *ALDOB*
C12	Smooth muscle cell	Stromal cell	*TAGLN*, *MYL9*, *MYH11*, *RGS5*
C13	Epithelial cell	Epithelial cell	*KRT7*
C14	NK cell	NK cell	*NKG7*
C15	Ciliated cell	Epithelial cell	*CST6*
C16	Smooth muscle cell	Stromal cell	*TAGLN*, *MYL9*, *MYH11*
C17	Secretory cell	Epithelial cell	*PDZK1IP1*, *ALDOB*
C18	Macrophage	Myeloid cell	*C1QC*, *AIF1*, *CD14*, *C1QA*
C19	Ciliated cell	Epithelial cell	*PSCA*
C20	B cell	B cell	*MS4A1*
C21	Spermatid	Spermatid	*TMEM190*, *TEX43*
C22	Spermatid	Spermatid	*TCP10L*
C23	Monocyte	Myeloid cell	*CTSS*, *PSAP*
C24	Epithelial cell	Epithelial cell	*KRT19*, *FABP1*
C25	Epithelial cell	Epithelial cell	*TOP2A*, *MKI67*
C26	Endothelial cell	Stromal cell	*VWF*
C27	Smooth muscle cell	Stromal cell	*TAGLN*, *RGS5*
C28	Spermatid	Spermatid	*TPPP2*
C29	Neutrophil	Myeloid cell	*S100A9*, *S100A8*
C30	Plasma cell	Plasma cell	*JCHAIN*
C31	Spermatid	Spermatid	*TMEM190*
C32	NKT cell	T cell	*CD3D*, *IL7R*
C33	Dendritic cell	Myeloid cell	*FCER1A*, *CLEC9A*
C34	FibSmo cell	Stromal cell	*MMRN1*
C35	B cell	B cell	*MS4A1*
C36	Mast cell	Myeloid cell	*CPA3*, *CMA1*
C37	Epithelial cell	Epithelial cell	*KRT19*
