# Supplementary material for: A reference single-cell regulomic and transcriptomic map of cynomolgus monkeys
Source: Nat Commun. 2022 Jul 13;13:4069. doi: 10.1038/s41467-022-31770-x (PMC9279386; doi:10.1038/s41467-022-31770-x)
Supplement: Supplementary file 2 — Reporting Summary [file 41467_2022_31770_MOESM2_ESM.pdf]

nature portfolio

Corresponding author(s): Dijun Chen

Last updated by author(s): Jun 15, 2022

Reporting Summary

Nature Portfolio wishes to improve the reproducibility of the work that we publish. This form provides structure for consistency and transparency in reporting. For further information on Nature Portfolio policies, see our Editorial Policies and the Editorial Policy Checklist.

Statistics

For all statistical analyses, confirm that the following items are present in the figure legend, table legend, main text, or Methods section.

n/a	Confirmed	
☐	☒	The exact sample size (n) for each experimental group/condition, given as a discrete number and unit of measurement
☒	☐	A statement on whether measurements were taken from distinct samples or whether the same sample was measured repeatedly
☐	☒	The statistical test(s) used AND whether they are one- or two-sided<br>*Only common tests should be described solely by name; describe more complex techniques in the Methods section.*
☒	☐	A description of all covariates tested
☒	☐	A description of any assumptions or corrections, such as tests of normality and adjustment for multiple comparisons
☐	☒	A full description of the statistical parameters including central tendency (e.g. means) or other basic estimates (e.g. regression coefficient) AND variation (e.g. standard deviation) or associated estimates of uncertainty (e.g. confidence intervals)
☐	☒	For null hypothesis testing, the test statistic (e.g. F, t, r) with confidence intervals, effect sizes, degrees of freedom and P value noted<br>*Give P values as exact values whenever suitable.*
☒	☐	For Bayesian analysis, information on the choice of priors and Markov chain Monte Carlo settings
☐	☒	For hierarchical and complex designs, identification of the appropriate level for tests and full reporting of outcomes
☒	☐	Estimates of effect sizes (e.g. Cohen's d, Pearson's r), indicating how they were calculated

Our web collection on statistics for biologists contains articles on many of the points above.

Software and code

Policy information about availability of computer code

Data collection	No software was used.
Data analysis	CellRanger (version 3.1.0)<br>DoubletFinder (version 2.0.3)<br>Seurat (version 4.0.0)<br>clusterProfiler (version 4.3.2.991)<br>UCell (version 1.99.7)<br>Monocle2 (version 2.99.3)<br>velocyto.R (version 0.6)<br>CellPhoneDB (version 2.1.1)<br>SCENIC (version 0.10.0)<br>arboreto (version 0.1.3)<br>igraph (version 1.2.8)<br>cellranger-atac (version 1.2.0)<br>ArchR (version 1.0.1)<br>MACS2 (version 2.2.7.1)

For manuscripts utilizing custom algorithms or software that are central to the research but not yet described in published literature, software must be made available to editors and reviewers. We strongly encourage code deposition in a community repository (e.g. GitHub). See the Nature Portfolio guidelines for submitting code & software for further information.

Data

Policy information about availability of data

All manuscripts must include a data availability statement. This statement should provide the following information, where applicable:

- Accession codes, unique identifiers, or web links for publicly available datasets
- A description of any restrictions on data availability
- For clinical datasets or third party data, please ensure that the statement adheres to our policy

The data that support the findings of this study have been deposited into CNGB Sequence Archive (CNSA) of China National GeneBank Data Base (CNGBdb) with accession numbers RNA: CNP0002427; ATAC: CNP0002441. Gene counts and metadata are available at Zenodo (https://zenodo.org/): DOI: 10.5281/zenodo.5881495. The Gene Expression Omnibus (GEO) accession numbers for scRNA-seq is GSE196792. The GEO accession number for scATAC-seq is GSE196791. The public dataset used in this study for cross-species comparisons between humans, mice, and monkeys can be accessed as described below: the human count matrix is available at https://figshare.com/articles/HCL_DGE_Data/7235471; the mouse count matrix is available at https://figshare.com/articles/MCA_DGE_Data/5435866.

Field-specific reporting

Please select the one below that is the best fit for your research. If you are not sure, read the appropriate sections before making your selection.

☒ Life sciences ☐ Behavioural & social sciences ☐ Ecological, evolutionary & environmental sciences

For a reference copy of the document with all sections, see nature.com/documents/nr-reporting-summary-flat.pdf

Life sciences study design

All studies must disclose on these points even when the disclosure is negative.

Sample size	Tissues were collected from 16 organs including trachea, spleen, stomach, kidney, uterus, tongue, testis, muscle, lung, liver, heart, colon, breast, bladder, adipose and aorta from two cynomolgus monkeys.
Data exclusions	No data were excluded from the analyses
Replication	Two cynomolgus monkeys were included in the analysis
Randomization	The experiments were not randomized
Blinding	The Investigators were not blinded to allocation during experiments and outcome assessment.

Reporting for specific materials, systems and methods

We require information from authors about some types of materials, experimental systems and methods used in many studies. Here, indicate whether each material, system or method listed is relevant to your study. If you are not sure if a list item applies to your research, read the appropriate section before selecting a response.

Materials & experimental systems

n/a	Involved in the study
☒	☐ Antibodies
☒	☐ Eukaryotic cell lines
☒	☐ Palaeontology and archaeology
☐	☒ Animals and other organisms
☒	☐ Human research participants
☒	☐ Clinical data
☒	☐ Dual use research of concern

Methods

n/a	Involved in the study
☒	☐ ChIP-seq
☒	☐ Flow cytometry
☒	☐ MRI-based neuroimaging

Animals and other organisms

Policy information about studies involving animals; ARRIVE guidelines recommended for reporting animal research

Laboratory animals	cynomolgus monkey, Macaca fasciculans.
Wild animals	This study did not involve wild animals.

Field-collected samples	This study did not involve samples collected from the field.
Ethics oversight	The cynomolgus monkey sample collection and research conducted in this study were approved by the Research Ethics Committee of the Changchun Biotechnology Development Co., Ltd. (Approval Number: 21001).

Note that full information on the approval of the study protocol must also be provided in the manuscript.
